# Investigation of the Drug Carrier Properties of Insoluble Cyclodextrin Polymer Microspheres

**DOI:** 10.3390/biom12070931

**Published:** 2022-07-02

**Authors:** Ádám Haimhoffer, Alexandra Vas, Gabriella Árvai, Éva Fenyvesi, László Jicsinszky, István Budai, Attila Bényei, Géza Regdon, Ágnes Rusznyák, Gábor Vasvári, Judit Váradi, Ildikó Bácskay, Miklós Vecsernyés, Ferenc Fenyvesi

**Affiliations:** 1 Department of Pharmaceutical Technology, Faculty of Pharmacy, University of Debrecen, Nagyerdei St. 98, H-4032 Debrecen, Hungary; haimhoffer.adam@pharm.unideb.hu (Á.H.); vas.alexandra96@gmail.com (A.V.); arvai.gabi11@gmail.com (G.Á.); rusznyak.agnes@pharm.unideb.hu (Á.R.); vasvari.gabor@pharm.unideb.hu (G.V.); varadi.judit@pharm.unideb.hu (J.V.); bacskay.ildiko@pharm.unideb.hu (I.B.); vecsernyes.miklos@pharm.unideb.hu (M.V.); 2 Doctoral School of Pharmaceutical Sciences, University of Debrecen, H-4032 Debrecen, Hungary; 3 Institute of Healthcare Industry, University of Debrecen, Nagyerdei St. 98, H-4032 Debrecen, Hungary; 4 Cyclolab Cyclodextrin R & D Laboratory Ltd., Illatos St. 7, H-1097 Budapest, Hungary; fenyvesi.e@cyclolab.hu; 5 Department of Drug Science and Technology, University of Turin, Via P. Giuria 9, 10125 Turin, Italy; ljicsinszky@gmail.com; 6 Faculty of Engineering, University of Debrecen, Ótemető Street 2-4, H-4028 Debrecen, Hungary; budai.istvan@eng.unideb.hu; 7 Department of Physical Chemistry, University of Debrecen, Egyetem Sqr. 1, H-4032 Debrecen, Hungary; benyei.attila@science.unideb.hu; 8 Institute of Pharmaceutical Technology and Regulatory Affairs, University of Szeged, Eötvös u. 6, H-6720 Szeged, Hungary; geza.regdon@pharm.u-szeged.hu

**Keywords:** insoluble cyclodextrin polymer, cyclodextrin beads, curcumin, estradiol-glow, complexation, drug delivery

## Abstract

The investigation of the usability of solid insoluble β-cyclodextrin polymers (βCDP) in micro-sized, controlled drug delivery systems has only recently attracted interest. Our aim was to form complexes with poorly soluble active pharmaceutical ingredients (APIs) with two types of βCDP for drug delivery applications. Solid insoluble cyclodextrin polymer of irregular shape (βCDPIS) and cyclodextrin microbeads (βCDPB) were used in the experiments. Morphology, surface area, size distribution and swelling capacity of carriers were investigated. We created complexes with two APIs, curcumin and estradiol, and applied powder X-ray diffraction, FTIR and thermal analysis (TGA/DSC) to prove the complexation. Finally, the dissolution, biocompatibility and permeation of APIs on Caco-2 cells were investigated. The size of the beads was larger than 100 µm, their shape was spherical and surfaces were smooth; while the βCDPIS particles were around 4 µm with irregular shape and surface. None of the polymers showed any cytotoxic effect on Caco-2 cells. Both carriers were able to extract curcumin and estradiol from aqueous solutions, and the dissolution test showed prolonged estradiol release. Caco-2 permeability tests were in accordance with the complexation abilities and dissolution of the complexes. This study offers useful data for further pharmaceutical applications of insoluble cyclodextrin polymers.

## 1. Introduction

Cyclodextrins (CD) are cyclic oligosaccharides composed of 6, 7 and 8 glucopyranose units which form a hydrophobic cavity with a hydrophilic surface. This structure is able to complex poorly soluble active pharmaceutical ingredients (API) and in most of cases can increase their permeability through biological barriers [1,2,3]. In addition to intermolecular forces between the host cyclodextrin and guest drug determined by the hydrophobicity of the guest molecule, the functional groups on the cyclodextrin and the derivatives with larger structures containing several cyclodextrins can significantly modify the complex-forming ability of carriers. The hydroxyl groups of the cyclodextrin ring at the C2, C3 and C6 positions are reactive, and the modification of the hydroxyl groups can help the extensive application of cyclodextrins in pharmaceutical development [4,5,6,7].

Cyclodextrin polymers offer new opportunities to broaden the scope of usability of drug delivery systems. [8]. The polymer structure may increase the possibility of the molecular interactions between the CD cavity and drug molecules [9]. Depending on the reaction conditions, it is possible to create soluble or insoluble polymers with the same reagents. The most common cross-linkers are epichlorohydrin, citric acid, and bifunctionalized activated acids as less toxic cross-linkers [3,8,10,11]. Whereas the main application of water soluble cyclodextrin polymers has been the solubilization of poorly soluble drugs [12,13,14,15], the insoluble types have been used mostly as a sorbent to remove pollution from contaminated water and to concentrate the analytes in analytical chemistry [9,16,17,18], although some human studies with insoluble polymers were also conducted in the early 1980s [19]. The size and shape of insoluble cyclodextrins affects their physical and biological properties also. The cyclodextrin polymers prepared in different shapes (pearl, foil, fiber or block) such as cyclodextrin beads were patented by Cyclolab in 1981 [20]. These beads were prepared by cross-linking with epichlorohydrin in the presence of polyvinyl alcohol, and the manufacturing process resulted in a regular spherical shape. In 1996 the insoluble CDP was modified with ionic groups and it was proved that the carboxymethyl polymer is a good sorbent for cationic API and the degree of carboxymethylation controlled the drug release [21]. In the same year Felméray et al. published on the wound healing ability of cyclodextrin polymer [22]. With the emerging green synthesis methods, the mechanochemical synthesis of cyclodextrin derivatives such as the cyclodextrin polymers was invented, and a solid, insoluble, β-cyclodextrin polymer (βCDPIS) was cross-linked with epichlorohydrin, such that the cyclodextrin microbeads were prepared in a planetary ball mill, under solventless conditions [23]. Due to the lack of the heterogeneous phases, the shape of the particles is irregular, and amorphous.

Despite the great potential drug carrier abilities of insoluble cyclodextrin polymers, there are limited numbers of studies which investigate this topic. By not being dissolved in the medium, their advantage can be the formation of a reservoir matrix. Compared to conventional matrix systems, these are smaller and contain the active ingredients in a molecularly dispersed/complexed manner. Furthermore, by complexing the active ingredient, they also have the benefits of cyclodextrins, thereby increasing the stability of the active ingredients, protecting the active ingredient from degradation or gastric acid damage, and being able to mask flavors and odors. Based on these assumptions, our aim was to study and compare the properties and the reservoir usability of βCDPIS and β-cyclodextrin microbeads (βCDPB) as micro-size, controlled drug delivery systems. For this purpose, two water-insoluble model drugs, curcumin (water solubility 3.12 mg/L) [24] and estradiol (water solubility 3.9 mg/L) [25] were used, and their complexation, liberation and biocompatibility were tested. The novelty of this work is that the insoluble cyclodextrin polymers had not yet been compared and tested systematically, and the results obtained reveal the possible application of the insoluble cyclodextrin matrix systems for oral drug delivery.

## 2. Materials and Methods

### 2.1. Materials

**Figure 1:**
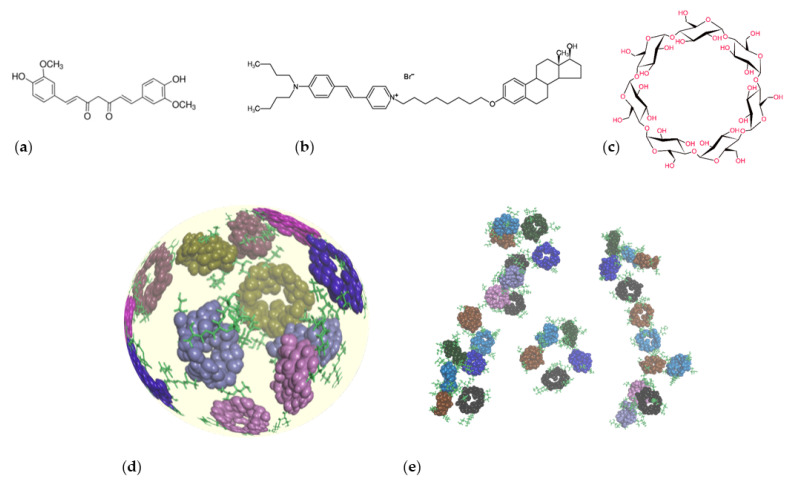
Chemical structures of curcumin (**a**), estradiol-glow (**b**), native β-cyclodextrin (βCD) (**c**), Cyclodextrin microbeads βCDPB (**d**) and β-cyclodextrin polymer of irregular shape βCDPIS (**e**).

The solid insoluble β-cyclodextrin polymer of irregular shape (βCDPIS) was synthesized at University of Turin (Turin, Italy), as reported earlier. Briefly at the first step, CD-sodium was formed, then epichlorohydrin cross-linking was performed. Reactions were carried out in a planetary ball mill (Retsch PM100 High Speed Planetary Ball Mill, Retsch GmbH, Haan, Germany), using a 50 mL stainless steel jar and mix of stainless steel balls (m = 44.1 g, in which ø = 5 mm, m = 28.1 g and 550 ø = 1–1.2 mm, m = 16.0 g) at 650 rpm [23,26]. Cyclodextrin microbeads (βCDPB) were made by Cyclolab Ltd. (Budapest, Hungary, Prod. No.: CY-2011). βCDP is prepared by cross-linking of βCD with epichlorohydrin in water, using NaOH as base, in the presence of polyvinyl alcohol at 70–80 °C [20,22]. This results in regular spherical beads of 0.1–0.3 mm diameter. Estradiol-glow (17-β-estradiol labeled with a low molecular weight biocompatible fluorophore) (Figure 1) was ordered from Jena Bioscience, and curcumin and the other reagents were from Sigma-Aldrich Ltd. (Budapest, Hungary).

### 2.2. Physical Properties of Insoluble Cyclodextrin Polymers

#### 2.2.1. Size Distribution

The samples were fixed on microscope slides by Fluoromount Aqueous Mounting Medium or in paraffin oil to prevent the moving of polymer, and were observed with a Zeiss AxioScope.A1 fluorescence microscope (HBO 100 lamp) (Carl Zeiss Microimaging GmbH, Göttingen, Germany). The average diameter of the particles was calculated by measuring the diameter of 20 particles by the help of ZEN 2012 v.1.1.0.0 software (Carl Zeiss Microimaging GmbH, Göttingen, Germany).

#### 2.2.2. Scanning Electron Microscopy (SEM)

Surface area exploration used a Hitachi Tabletop microscope (TM3030 Plus, Hitachi High-Technologies Corporation, Tokyo, Japan) in high-resolution mode. The samples were attached to a fixture with a double-sided adhesive tape containing graphite. Before SEM examination gold-sputtered coating was not deposited on the surface of the samples, as the instrument is suitable for the direct investigation of the specimens without any surface pre-treatments. The measurement requires a vacuum and low, 5kV accelerating voltage. Magnifications were 60×, 250×, 500× and 15,000×.

#### 2.2.3. Swelling Capacity

An Enslin device (manufactured in-house) was used to determine the swelling properties of polymers in three types of medium, namely 30% ethanol, 10% DMSO and water (pH 6.5) [27,28]. In ethanol and DMSO solutions, the swelling capacity plays an important role in complex formation, while swelling in water is also associated with drug release in the human body. Samples (100 mg) were placed on a filter of the apparatus and the volume of absorbed water was read at specific time points at 10, 20, 30, 60, 120, 180, 240, 300, 360, 480, 600 and 900 *s*. Swelling capacity of polymers was expressed by the volume of water uptake per gram of polymer (mL/g).

### 2.3. Complexation Properties of Insoluble Cyclodextrin Polymers

#### 2.3.1. Complexation Study and Preparation of Complexes

To study the complexation of the cyclodextrin polymers with the applied APIs, curcumin and estradiol-glow were precisely measured and dissolved in 96% ethanol and pure DMSO, respectively, to create 1 mM stock solutions. These solutions were diluted with water and the applied solvents to reach the final concentrations of APIs (10, 40, 100, 200 µM) in 30% ethanol or 10% DMSO solutions, respectively. For the complexation study, 20 mg insoluble cyclodextrin polymer carriers were added to the solutions, and the suspensions were stirred at room temperature up to 24 h for the formation of complexes (βCDPIS-est, βCDPB-est, βCDPIS-cur, βCDPB-cur). After centrifugation (5000 rpm, 10 min), the API content of supernatants were determined by UV-VIS (curcumin λ_abs_ = 430 nm, Shimadzu UV-1900 spectrophotometer, Kyoto, Japan) and fluorescence spectroscopy (estradiol-glow λ_exc_ = 544 nm, λ_em_ = 612 nm; FLUOstar OPTIMA microplate reader BMG LABTECH, Offenburg, Germany), and the complexed amount of APIs were calculated. Loading efficiency was calculated from the extraction curve of API by the following method [29]:

(1) $$\mathrm{API}\;\mathrm{loading}\;\mathrm{efficiency}\;\left(\%\right)=\frac{\mathrm{Weight}\;\mathrm{of}\;\mathrm{extracted}\;\mathrm{API}\;}{\mathrm{Weight}\;\mathrm{of}\;\mathrm{initial}\;\mathrm{API}}\times 100$$

Preparation of complexes: for further experiments, 200 µM API solutions were used to create complexes with the method described above. After the complexation process, the sedimented parts, namely the complexes, were freeze-dried using a freeze drier, and the complex were kept in a freezer at −20 °C. The API loading capacity was calculated by the following equation:

(2) $$\mathrm{API}\;\mathrm{loading}\;\mathrm{capacity}\;\left(‰\right)=\frac{\mathrm{Weight}\;\mathrm{of}\;\mathrm{extracted}\;\mathrm{API}\;}{\mathrm{Weight}\;\mathrm{of}\;\mathrm{final}\;\mathrm{product}}\times 1000$$

#### 2.3.2. FTIR Analysis

The FTIR ATR spectra experiments were performed on a PerkinElmer Spectrum Two instrument (LiTaO3 detector, diamond window) by Spectrum IR v10.2 software, using 64 scans at 1 cm^−1^ resolution of 0.25 cm^−1^ data collection interval. Corrections of environmental CO_2_ and H_2_O used the software’s built-in method. Evaluation of spectra used SpectraGryph 1.2.16d software (Software for optical spectroscopy by Dr. Friedrich Menges, Oberstdorf, Germany, http://spectroscopy.ninja (accessed on 15 January 2021)).

#### 2.3.3. Powder X-ray Diffraction (PXRD)

To analyze the crystal form of APIs and polymers, physical mixtures and freeze-dried complexes were prepared, and ultrapure API was used as a control. The finely powdered sample was fixed onto a Mitegen MicroMeshes sample holder (MiTeGen Co., Ithaca, NY, USA) with a minimal amount of oil. Powder diffraction data of the samples with Debye Scherer geometry were collected using a Bruker-D8 Venture (Bruker AXS. GmbH, Karlsruhe, Germany) diffractometer equipped with INCOATEC IμS 3.0 dual (Cu and Mo) sealed tube micro sources (50 kV, 1.4 mA). A Photon 200 Charge-integrating Pixel Array detector and CuKα (λ = 1.54178 Å) radiation was applied. Several frames were collected with various detector-sample distances in phi scan mode. Data collection and integration was performed using the APEX3 and DiffracEva software (Bruker AXS Inc., Madison, WI, USA, Version 4.2.2.3), respectively.

#### 2.3.4. Thermoanalytical Methods (TGA/DSC)

The thermal analysis (thermogravimetric analysis-TGA and differential scanning calorimeter-DSC) of the samples was carried out with a Mettler-Toledo TGA/DSC1 instrument (Mettler-Toledo GmbH, Greifensee, Switzerland). Quantities between 3 mg and 10 mg of samples were placed in a closed aluminum crucible (Mettler-Toledo GmbH, Greifensee, Switzerland) with a volume of 40 μL. The temperature range was between 25–500°C, and the applied heating rate was 10°C/min. Nitrogen atmosphere was used (cell gas: 50 mL/min, method gas: 70 mL/min). With the objective of comparing the curves, the results were normalized to sample weight and the evaluation of the curves was acquired with STAR^e^ Software (Mettler-Toledo GmbH, Greifensee, Switzerland).

### 2.4. MTT (3-(4,5-Dimethyl-2-thiazolyl)-2,5-diphenyl-2H-tetrazolium bromide) Assay

The cytotoxic effects of cyclodextrin carriers were evaluated using MTT test. Caco-2 cells were seeded in 96-well plates at a density of 10,000 cells/well. After 3 days, the medium was removed, and the cells were incubated for 30 min with the suspensions of carriers (0–25.0 mg/mL). The samples were then removed, and a 0.5 mg/mL MTT (3-(4,5-Dimethyl-2-thiazolyl)-2,5-diphenyl-2H-tetrazolium bromide) solution (in phosphate-buffered saline—PBS) was added to each well. The plates were incubated for 3 h at 37 °C, then the MTT solution was removed and 0.1 mL isopropanol:1 M hydrochloride acid (25:1) was added to each well to dissolve the formed formazan crystals. The absorbance of dissolved formazan was measured at 570 nm and the background was measured at 690 nm by Thermo-Fisher Multiskan Go (Thermo-Fisher, Waltham, MA, USA) microplate reader.

### 2.5. In Vitro Dissolution Test

A modified dissolution test was elaborated for the investigation of the dissolution of APIs from the polymer carriers due to the small amount of samples and the low water solubility of curcumin and estradiol. An amount of 20 mg complex was suspended in 1.5 mL of fetal bovine serum (FBS), which was applied as the dissolution medium in the tests. The experiments were performed at 37 °C with continuous shaking at 100 rpm. Samples of 0.50 mL were withdrawn after 15 min, 30 min, 60 min and 120 min. Three parallel measurements were performed with each complex, and the released amount of curcumin and estradiol was determined by the following method.

In the case of curcumin, samples were purified from protein by adding acetonitrile to the samples (2:1 volume ratio). The obtained solutions were analyzed using a HPLC system (Merck-Hitachi ELITE with photodiode array detector (DAD)). The column was an Agilent HC-C18(2) (150 × 4.6 mm), kept at 40 °C, and the DAD was set at 430 nm. The mobile phase was acetonitrile and 2% acetic acid solution (4:6), and 1.0 mL flow rate was used. The analyses were performed with EZChrom Elite software TM (Hitachi, Tokyo, Japan), which was also used for collecting and processing the data. 10 μL standard solution and purified samples were injected.

Estradiol was measured directly from the dissolution medium by fluorescence spectroscopy (estradiol-glow λ_exc_ = 544 nm, λ_em_ = 612 nm; FLUOstar OPTIMA microplate reader BMG LABTECH, Offenburg, Germany).

### 2.6. In Vitro Permeability Study

In vitro permeability studies used Transwell^®^ polycarbonate filters (area: 1.12 cm^2^, pore size: 0.4 µm) (Corning, Glendale, Arizona, USA ) to grow Caco-2 monolayers. Studies were started with the initial seeding of 250,000 cells/transwell and when the transepithelial electrical resistance (TEER) reached 300 Ωcm^2^, the monolayers were used in the permeability experiments. Samples of complexes of 20 mg each, made by complexation with 200 µM API solutions, were dispersed in Fetal Bovine Serum (FBS) and placed on the apical surface of the monolayers. During the experiments, the basal chamber also contained FBS. Control samples were treated with only estradiol-glow or curcumin, respectively.

In the case of estradiol-glow, samples were taken from the basal sides of the cell layers at specific time points (0,5 h, 1 h, 2 h, 4 h, 8 h, and 24 h) and the permeated amount of estradiol-glow was measured with the fluorescence microplate reader method as mentioned above.

In some permeability experiments, the estradiol-glow cellular uptake was examined by the following method. Caco-2 cells were previously seeded onto sterile round glass coverslips and placed under cell-free Transwell^®^ polycarbonate filters, during the permeability experiments. In this study, we sought to examine whether estradiol-glow is able to reach the cellular target after liberation and by free diffusion from the insoluble complex, and can accumulate in the more lipophilic intracellular environment. After 2 h of the test, cells were washed with Hank’s Balanced Salt Solution (HBSS) fixed with 3% paraformaldehyde solution, and cell nuclei were stained in 300 nM 4′,6-Diamidino-2-phenylindole dihydrochloride (DAPI) solution at room temperature for 15 min. Finally, the samples were mounted onto glass microscopic slides. The uptake of estradiol-glow was investigated by fluorescence microscopy using a Zeiss Axioscope A1 fluorescence microscope (HBO 100 lamp) (Carl Zeiss Microimaging GmbH, Göttingen, Germany) with the following filters: DAPI: excitation G 365 nm, emission BP 445/50 nm; and estradiol-glow: excitation BP 546/12 nm, emission BP 575–640 nm. PBS without estradiol-glow was used as negative control.

In the case of curcumin permeability studies, after 24 h of incubation of monolayers with the complexes, samples from the basal chambers were collected and analyzed as described in Section 2.5.

The apparent permeability coefficient (P_app_) of chrysin was calculated using the following equation [30]:

(3) $$P_{\mathrm{app}}=\frac{dQ}{dt}\times \frac{1}{\left(C_{0}\times A\right)}$$

where P_app_ is the apparent permeability coefficient (cm/s); *dQ*/*dt* is the permeability rate of substances (mol/s); *C*_0_ is the initial concentration of the substances in the upper compartment (mol/mL); and *A* is the surface area of the membrane (cm^2^).

### 2.7. Statistical Analysis

Mean values of different groups were compared by one way ANOVA using SigmaStat 3.5 software (Systat Software, Inc., San Jose, CA, USA). Data are presented as means ± SD, and differences were considered significant at *p* < 0.05.

## 3. Results

### 3.1. Physical Properties of Insoluble Cyclodextrin Polymers

#### 3.1.1. Size Distribution

**Figure 2:**
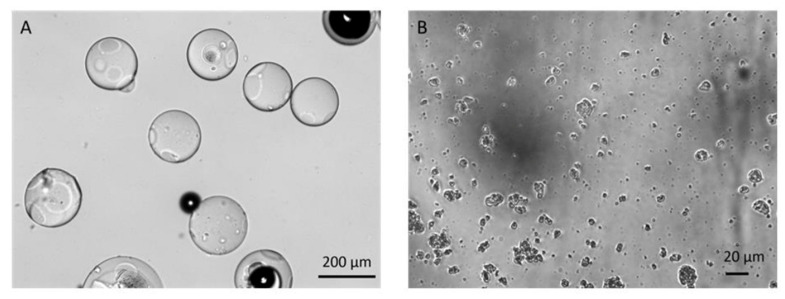
Light microscopy images of (**A**) βCDPB and (**B**) βCDPIS carriers in water.

The light microscopy pictures of carriers show the different morphologies of the βCDPB and βCDPIS, despite the cross-linker and monomer being identical (Figure 2.). While the former has smooth surfaces with a spherical shape and uniform sizes, the latter has a smaller, aggregated structure, and shows high heterogeneity of size distribution.

The size of the beads is greater by two orders of magnitude than βCDPIS particles as presented in Table 1. Beads swelled rapidly in water, and after 15 min their size increased significantly (*p* < 0.001) compared to the beads kept in paraffin oil (see also Supplementary Figure S1). Both swelling and particle size reduction can increase the accessible surface of particles, which can increase the quality and quantity of complexation.

#### 3.1.2. SEM

**Figure 3:**
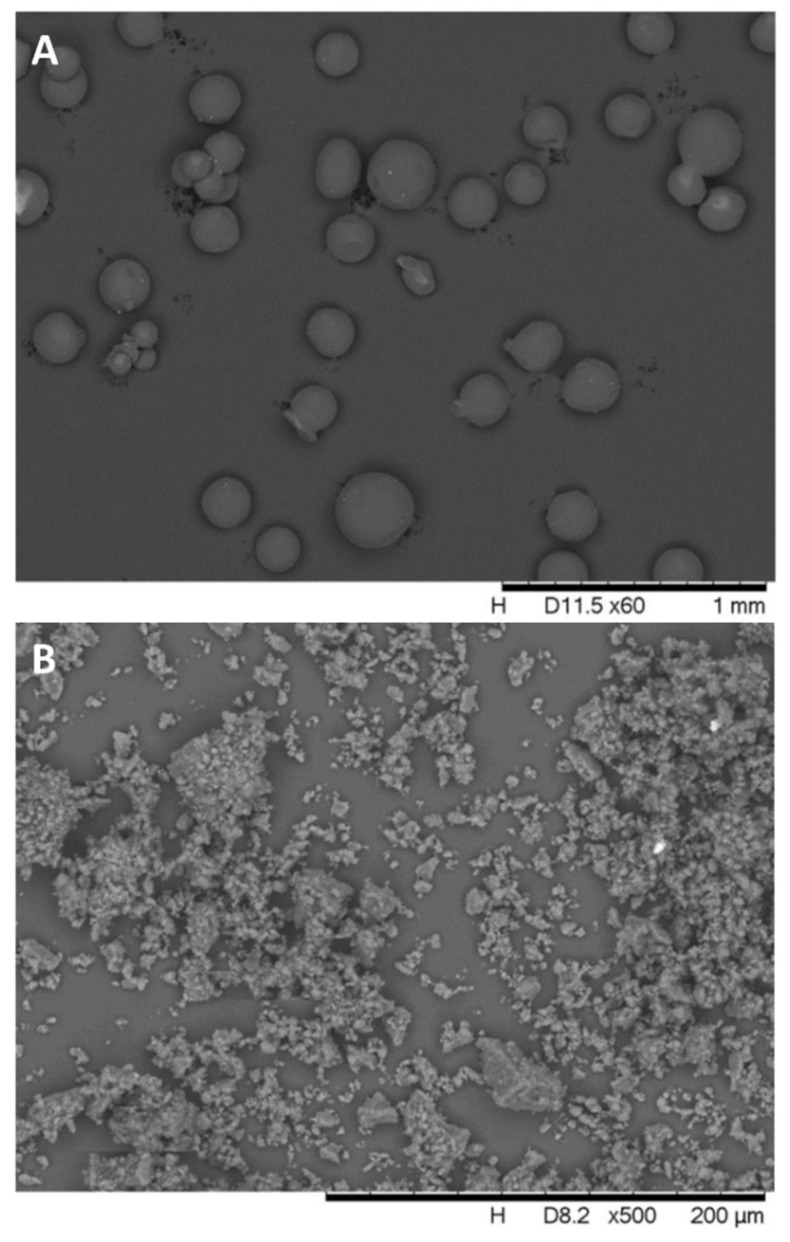
Scanning electron microscopy images of (**A**) βCDPB and (**B**) βCDPIS carriers.

The SEM confirmed the light microscopy results presented in Figure 3. The shape of beads is roundish. The surface showed little roughness at larger magnification, and its surface can still be called smooth. No visible micro pores or channels could be detected on the surface of beads at this magnification. The image of βCDPISs confirmed the heterogeneity of particle size distribution. The surface is rugged, and some pores and channels can be found between the particles.

#### 3.1.3. Swelling Capacity

**Figure 4:**
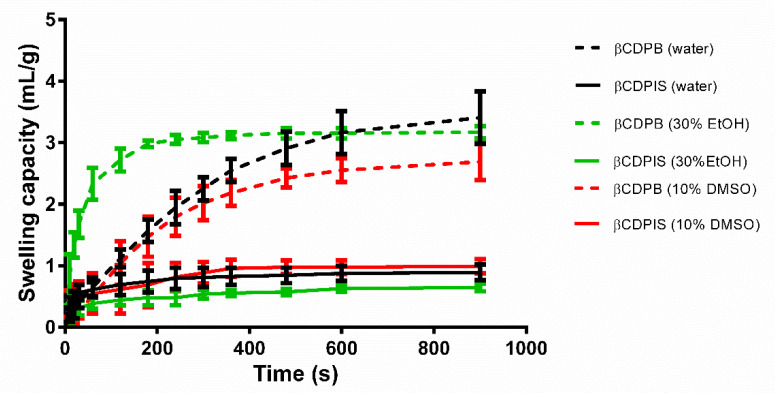
Swelling capacity of carriers in water, 30% Ethyl alcohol, and 10% DMSO solution (data are presented as means ± SD, *n* = 3).

The determination of swelling of insoluble polymer particles was performed prior to complexation. Measuring the liquid absorption of cyclodextrin polymer particles ensures information about the structural changes of particles in different solvents. βCDPB has different capacity and swelling kinetics in water, 30% ethyl alcohol, and 10% DMSO solution compared to βCDPIS. In 30% ethanol solution, the fluid uptake was significantly steeper than the other two solvents; and 10% DMSO showed the lowest (2.7 mL/g) capacity after 15 min (Figure 4). In the case of βCDPIS, the best swelling capacity was obtained in 10% DMSO, reaching 1.0 mL/g liquid uptake up to 15 min. The saturation curve of 30% ethanol was below that of water, and the final point was 0.6 mL/g, despite its co-solvent ability. The curves revealed that the βCDPIS was wetted well, but it did not swell as expected.

### 3.2. Complexation Properties of Insoluble Cyclodextrin Polymers

#### 3.2.1. Complexation Study

**Figure 5:**
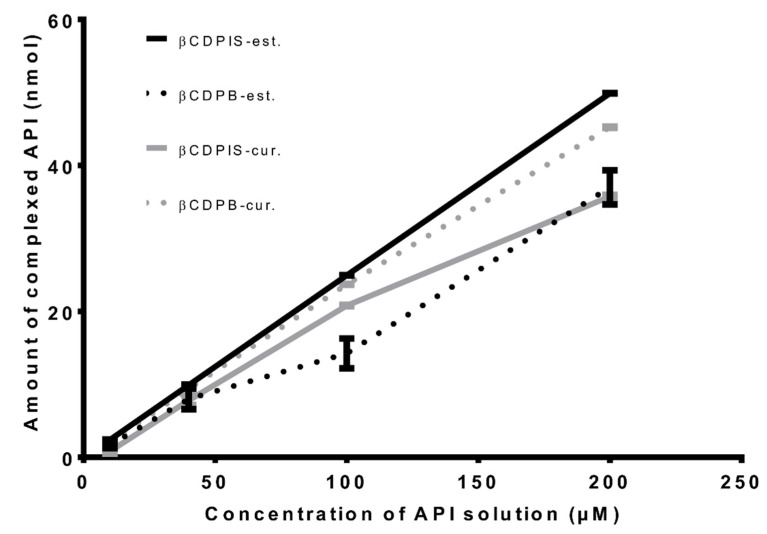
Complexation study of curcumin (cur) and estradiol-glow (est) from aqueous solutions by 20 mg βCDPIS and βCDPB (data are presented as means ± SD, *n* = 3). ΒCDPIS-est: estradiol-glow complexed with ΒCDPIS; ΒCDPIS-cur: curcumin complexed with ΒCDPIS; βCDPB-est: estradiol-glow complexed with βCDPB; βCDPB-cur: curcumin complexed with βCDPB.

The complexation of curcumin and the fluorescent estradiol derivative estradiol-glow was studied in the solutions of these drugs. For this, a fixed amount of the polymers (20 mg) was incubated with the API solutions in increasing concentrations, and the amount of complexed APIs was determined as described in the Methods, Section 2. The polymers efficiently extracted the APIs from aqueous solutions. There was a linear relationship between the concentration of APIs in solution and the amount of complexed drugs. Up to 200 µM, the polymers were not saturated with the applied quantity. The βCDPIS showed lower affinity to curcumin, but the curve of estradiol-glow runs higher than in the case of βCDPB, and shows linearity as can be seen in Figure 5. The API loading efficiencies were higher than 70% in each case, which is a fairly high value in itself, but can be increased further to 90% by selecting the better carrier. βCDPIS almost completely complexed the available estradiol-glow quantity from the solution (Table 2).

#### 3.2.2. FTIR Analysis

**Figure 6:**
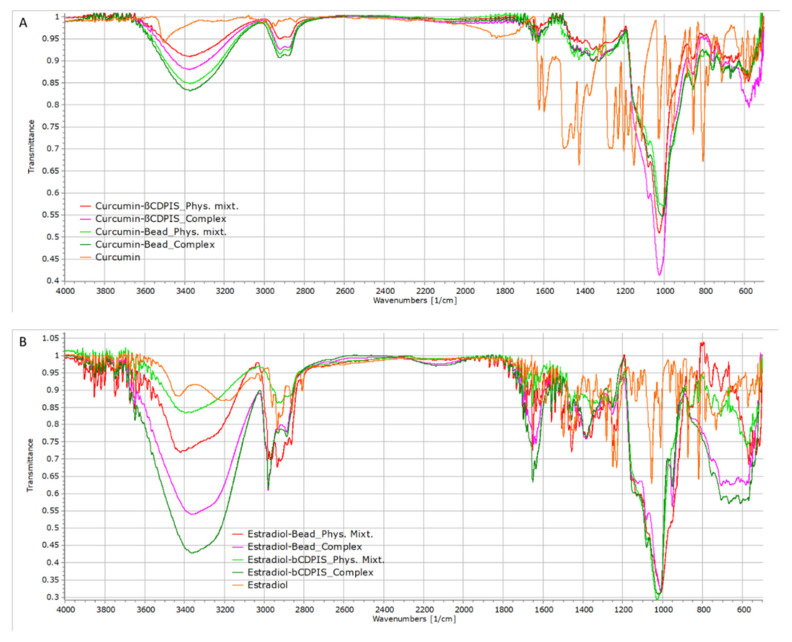
IR spectra of pure APIs, physical mixtures, and complexes with (**A**) curcumin and (**B**) estradiol.

IR spectra registration used 1 cm^−1^ resolution, which at least in theory is suitable for seeing the complexation effects on the characteristic IR-bands in Figure 6. Despite the high resolution, as a result of the numerous overlapping signals of the CD polymers and guests, the IR band shifts generally remained within resolution limits by the higher number of similar vibration regions of functional groups in the CD than in the guest molecules. The IR band assignments used the recorded spectra of pure compounds and data found in the literature [31,32,33,34,35,36,37].

Inspection of the signal overlapping effect, synthetic ~1:1 physical mixture spectra were also created using the measured spectra of pure chemicals by proportional addition of the components. The comparison of the simulated and recorded physical mixtures showed significantly less identifiable IR bands in the recorded spectra. The bead polymer, due to its hardness, remained practically intact during the sample preparation. The compounds were distributed inhomogeneously on the surface of the βCDP and between the polymer particles, while the significantly smaller βCDPIS particles formed a homogeneous dispersion. This incomplete mixing with βCDP resulted in more peak appearances in the recorded physical mixture spectra of βCDPB than in the βCDPIS, as seen in Figures S4 and S6 of the Supplementary Material.

The mechanochemically synthesized insoluble polymer contains fewer hydroxyl groups than the bead polymer because hydrolysis of epichlorohydrin during synthesis is limited. The nearly relative identical OH/H-bond (3200–3500 cm^−1^) and water (1610–1660 cm^−1^) regions (Figure S3) are in contrast to the C-O/C-C (1000–1100 cm^−1^) region which is significantly larger in βCDPIS. The complex preparation increased the water content of the polymers in complexes, as shown by the higher intensities of the hydrogen bond OH region (3200–3500 cm^−1^). Interestingly, a phenolic OH signal (>3580 cm^−1^) appeared in the βCDP, suggesting some residual uncomplexed curcumin on the βCDP surface. The similar signal of estradiol completely disappeared in the physical mixture of βCDP and complexes. The higher water content of complexes resulted in a further signal reduction in the 1600–1700 cm^−1^ region, where the water signal suppresses some aromatic bands. While the aromatic vibrations are visible in the spectrum of the synthetic physical mixture, these signs of guest molecules are often invisible in the recorded spectra of the physical mixture, and even more so in the complex. Because this region overlaps with the aromatic vibrations of both guest molecules, the effect of complexation to the aromatic moieties remained hidden. Although the C-H stretching bands (2750–3000 cm^−1^) in the estradiol cases showed significant peak shifts, these moved below the twin peaks. The curcumin samples showed significant peak shifts (3–20 cm^−1^), indicating the modification of the H-bond networks in the polymer.

The conjugated dienone signals are weak in the neat curcumin (1684 cm^−1^), and they completely disappeared in both the physical mixtures and complexes.

A minimal peak movement is in the aromatic C-C ring stretching region (1410–1609 cm^−1^) but with only a few significant differences between the physical mixtures and complexes. This region overlaps with the CH2/CH3 bending region, and although the functional group assignment is ambiguous, the host-guest interaction is seen. In complexes, the out-of-plane aromatic C-H vibrations (750–870 cm^−1^) showed some significant changes in the case of estradiol, but the band assignment is at least as ambiguous as in the case of higher wavenumbers. In the case of curcumin, these shifts are within the experimental errors or negligible.

Although both guests have methyl (curcumin)/methylene (estradiol) groups, only some of the CH2/CH3 bending and CH3-rocking vibrations (1300–1400 cm^−1^) showed significant changes, suggesting that their interaction with the cyclodextrin polymeric network, or possibly that cross-linking groups are weak.

The C-O/C-C stretching vibrations (1000–1200 cm^−1^) showed the 4–20 cm^−1^ peak shifts, though it was impossible to identify which part of the guest molecules was involved in the complex formation due to the many similar functional groups.

The aromatic regions of estradiol are affected significantly in both polymer complexes, suggesting a partial complexation of the A-ring of the steroid. Although vibrations of the aromatic moieties of curcumin showed minimal changes, which is also the primary target molecule for complexation, the ortho-position of OH/OCH_3_ groups is disadvantageous for full inclusion. The insignificant shifts of the aromatic regions also point out the rigidness of the polymeric structures, and the superposition of the complexed and uncomplexed signals results in minimal differences only in the peak locations.

All the recorded and simulated IR spectra with the peak assignment are in the Supplementary Material (Figures S2–S7, Tables S1–S3).

#### 3.2.3. PXRD

**Figure 7:**
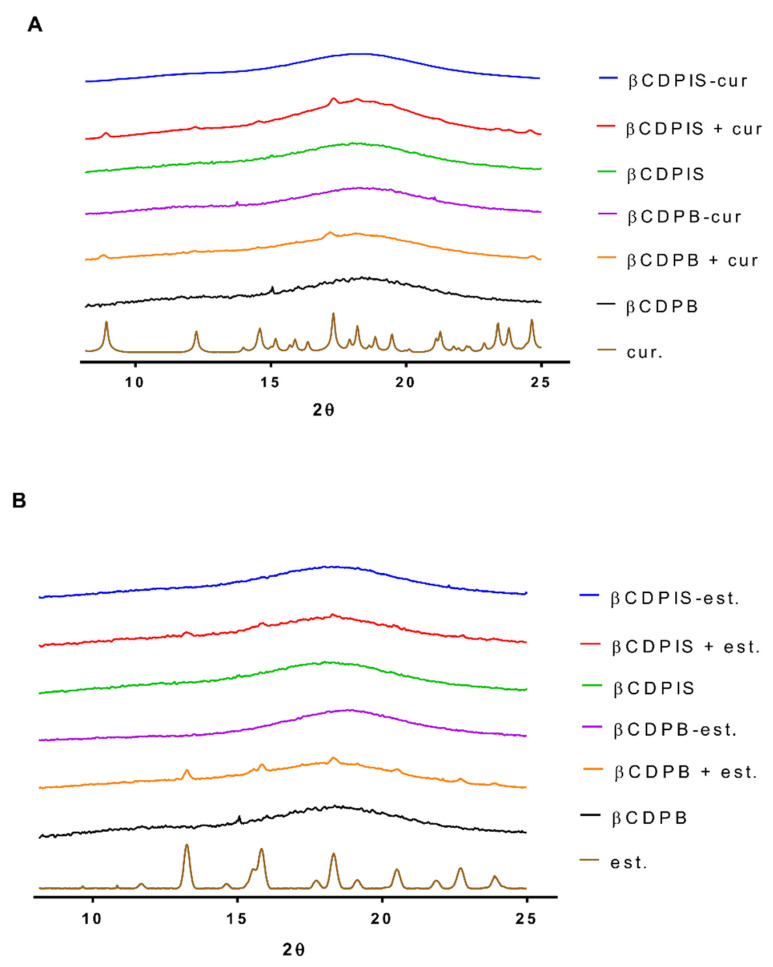
X-ray diffraction patterns of pure APIs, physical mixtures, and complexes. (**A**) Complexes and physical mixtures of curcumin (cur) with polymers. The crystal form of curcumin disappeared from the scatters after the complexation. (**B**) Changes in the crystallinity of estradiol (est) in different complexes. The nomenclature of complexes and physical mixtures contains “−“ and “+” symbols respectively.

The X-ray diffraction patterns of curcumin and estradiol, physical mixtures of APIs and carriers, and API-carrier complexes are shown in Figure 7. The specific, sharp peaks of curcumin appeared at 8.907°, 12.160°, 14.579° and 17.339° indicating crystal forms [13]. The crystalline scatters of estradiol were observed at 13.3°, 15.8°, 18.3°, 20.5° and 22.8° [38]. The amorphous carriers (βCDPB and βCDPIS) showed broad bands, but peaks of curcumin and estradiol crystals were also found on the curves of physical mixtures (βCDPIS + cur, βCDPB + cur and βCDPIS + est, βCDPB + est). After the complexation, these crystalline characters decreased or disappeared from the curves. The lack of crystalline structures shows the interaction between the cyclodextrin polymers and APIs.

#### 3.2.4. Thermoanalytical Investigations (TGA/DSC)

**Figure 8:**
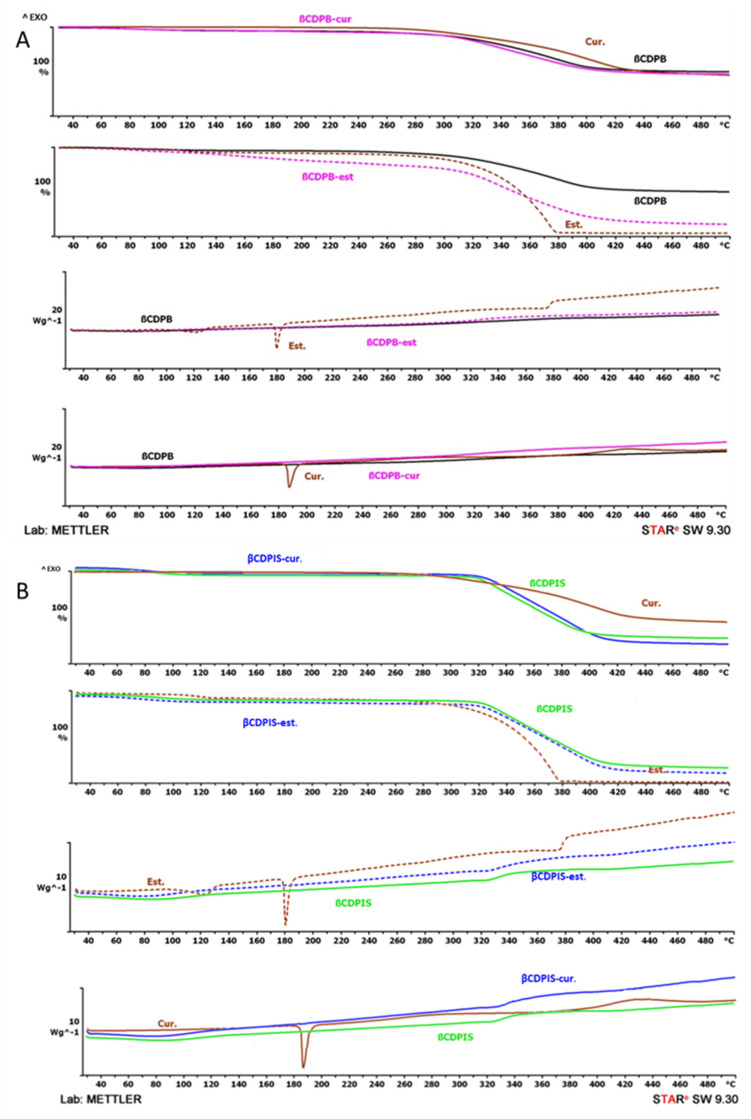
TGA (upper two sections of subfigures A and B) and DSC (lower two sections of figures A and B) analysis of native polymers, APIs and their inclusion complexes with βCDPB (**A**) and βCDPIS (**B**).

Thermal analysis (TGA and DSC) was done on samples to identify the changes caused by temperature change. It was performed on pure insoluble cyclodextrin polymers, curcumin and estradiol, and their inclusion complexes. Results were plotted in Figure 8 in the temperature range of 25 °C to 500 °C. The DSC curves show that pure curcumin and estradiol have characteristic endotherm peaks at 186.17 and 179.60 °C, respectively, showing their melting, while these peaks disappeared in the case of the complexes. This indicates that both curcumin and estradiol are in an amorphous form in the complexes. Each of the materials exhibited a weight loss due to decomposition above 320 °C.

#### 3.2.5. MTT Assay

**Figure 9:**
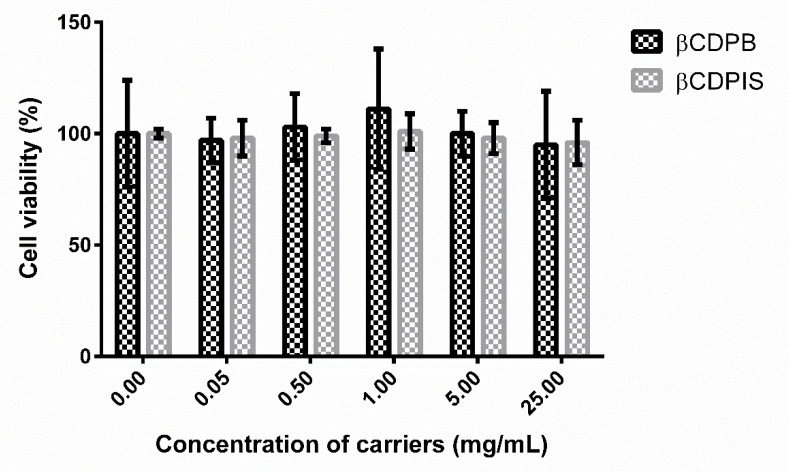
MTT assay results with increasing concentration of βCDPB (black) and βCDPIS (gray) after 30 min incubation (data are presented as means ± SD, n = 5).

The MTT assay was used to prove the biocompatibility of carriers. The highest concentration of the carriers was 25.0 mg/mL, that settled quickly as a concentrated suspension. Cyclodextrin polymers did not cause significant decrease (*p* > 0.05) in cell viability compared to the untreated control, indicating that cell damage was not observed after 30 min incubation as shown on Figure 9. Therefore, it can be said that the carriers did not have a toxic effect even at very high concentrations and do not contain any toxic substances that would dissolve from them after preparation.

#### 3.2.6. In Vitro Dissolution Study

**Figure 10:**
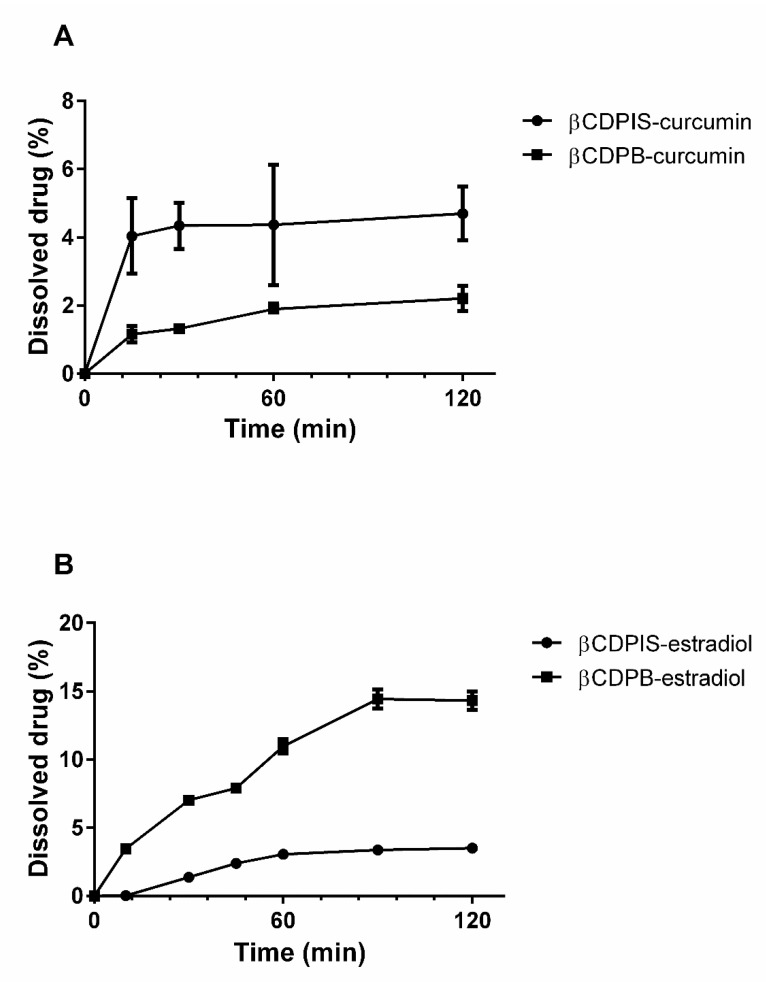
Dissolution profile of curcumin (**A**) and estradiol (**B**) complexes.

Significant differences could be observed among the dissolution profiles of the complexes (Figure 10). In the case of curcumin, approximately 4% of curcumin dissolved from βCDPIS complex after 120 min, with fast dissolution in the first 15 min; while in the case of βCDPB, less than 2% curcumin dissolved after 2 h. The dissolution profile of βCDPB-curcumin complex showed a slowly increasing tendency. Interestingly, the dissolution test of estradiol complexes showed opposite results. Higher estradiol concentration released into the medium from βCDPB-estradiol complex and reached 15% after 2 h. βCDPIS delivered only 3% estradiol from the complex into the medium.

#### 3.2.7. In Vitro Permeability Study and Cellular Uptake of Estradiol-Glow

**Figure 11:**
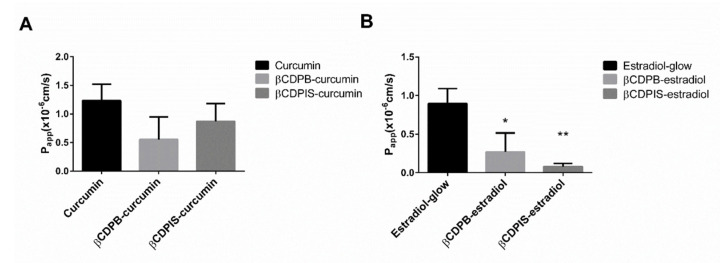
In vitro permeability study of Curcumin (**A**) and Estradiol-glow (**B**) on Caco-2 monolayers (data are presented as means ± SD, n = 3, P_app_—apparent permeability coefficient, * *p* < 0.05 for βCDPB and ** *p* < 0.01 for βCDPIS compared to control estradiol-glow treatment).

Due to the low drug release from the complexes, in vitro permeability studies were carried out for up to 24 h on Caco-2 monolayers (Figure 11). Even though βCDPIS caused a higher P_app_ of curcumin than βCDPB, curcumin penetrated better through the cell layer from the control (curcumin-only treatment) than from the complexes, and these differences were not significant (*p* > 0.05). In the case of estradiol, the P_app_ of estradiol from the βCDPB carrier was not significantly better than from βCDPIS. Estradiol-glow had significantly better permeation from the control solution (estradiol-glow treatment only) than from the complexes (*p* < 0.05 for βCDPB and *p* < 0.01 for βCDPIS).

**Figure 12:**
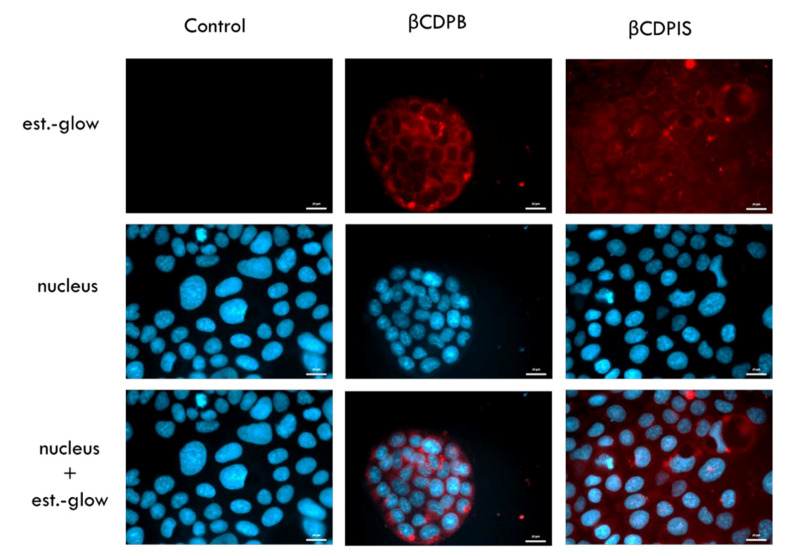
Fluorescence microscopy images of estradiol-glow uptake after 2 h of incubation on Caco-2 cells. Estradiol-glow accumulated in the cytoplasm (red) after incubation indicating that it liberated from the carrier and reached the cellular target. Control: cells were treated with PBS without estradiol-glow. Blue: cell nuclei, red: estradiol-glow. Scale bar: 20 µm.

After 2 h of incubation of the complexes on cell-free Transwell^®^ inserts, estradiol-glow was taken up into Caco-2 cells which were placed into the basal chamber (Figure 12), under the membrane. The API could be liberated from the complexes and by free diffusion penetrated into cells reaching the cytoplasm and the site of action. Lipophilic estradiol-glow appeared and accumulated in the cytoplasm and visualized the morphology of the cells, while the surrounding environment remained a black background.

## 4. Discussion

**Figure 13:**
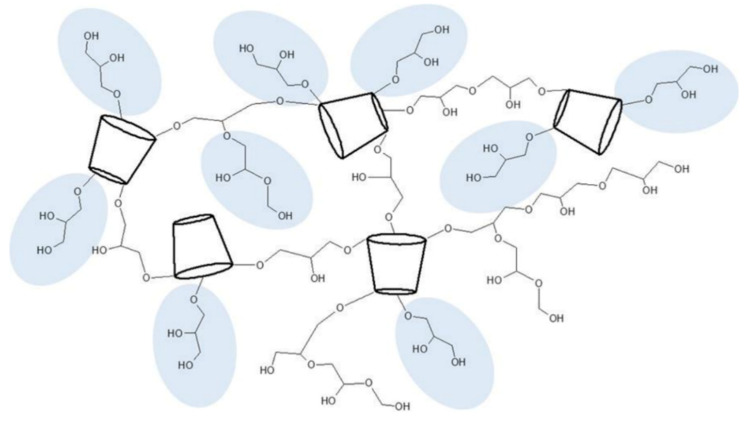
Scheme of the structures of βCDPs. The dihydroxypropyl side-chains (marked in blue) are formed in aqueous reaction systems, but are missing in the polymer prepared under solventless conditions.

In the last few years, more and more studies have been published on cyclodextrin polymers. Some of these have researched water-insoluble ones, but most of them focused on their application in environmental protection. Our aim was to investigate two insoluble cyclodextrin polymers of similar structure as drug carriers, which can be considered also as reservoir matrix systems. Due to their particle size and water insoluble nature, their most adequate application could be that of oral drug administration carrying APIs of low solubility or poor absorption from the gastrointestinal tract. We chose two chemically different model substances to test their interactions with βCDPIS and βCDPB. Both curcumin and estradiol are administered orally, having low solubility; and curcumin also has poor absorption and bioavailability [39]. Both polymers were prepared by cross-linking βCD with epichlorohydrin, but under different conditions. The traditional two-phase reaction was used to prepare βCDPB applying toxic solvents which are carefully removed during a laborious purification step, or a solventless green technology is applied for the production of βCDPIS. Although their chemical nature is similar, the size and shape of particles shows large differences: the beads are bigger than βCDPIS particles. The latter is produced by ball milling where the simultaneous synthesis and milling results in obviously small particle size. The βCDPB has a smooth surface and spherical shape, while the βCDPIS particles are rugged and irregular. The porous structure of the bead is non-visible at the applied magnification, and it has the ability to swell several times its weight probably because of the hydrophilic network structure. The bead polymer absorbs 4–5 times their weight from water and aqueous solutions (e.g., biological liquids) [22]. The βCDPIS showed lower swelling due to a more cross-linked structure [26]. Comparing the swelling capacity of beads, it is less in both 30% ethanol and 10% DMSO than in water; while βCDPIS swelled the most efficiently in 10% DMSO, although there was no high difference in the swelling in the studied solvents. The preparation of the beads by the traditional emulsion method results in dihydroxypropyl side-chains due to the partial hydrolysis of the cross-linking epichlorohydrin. The extra hydroxyl groups in theses dihydroxypropyl side chains cause enhanced hydrophilicity and enhanced swelling in more hydrophilic solvents. In the solventless preparation of βCDPIS, hydrolysis hardly occurs, resulting in more cross-linked structures of lower hydrophilicity and reduced swelling (Figure 13).

The better swelling of beads caused higher loading efficiency for the curcumin complexation, shown by the higher affinity of curcumin to microbeads. The opposite trend for estradiol-glow can be attributed to the less hydrophilic character of the network. Both APIs selected for these studies form 2:1 host:guest complexes. They were selected to utilize the close proximity of the CD rings able to cooperatively bind these guest molecules. Postulating that both carriers have approximately the same cyclodextrin content, the differences can be explained by the different accessibility of the cyclodextrin cavities and by the different properties of the network. The secondary cavities formed by the network contribute to the binding of the two model APIs to a different extent [9].

In this study, a very simple complexation method was applied to the insoluble polymers, which were incubated with the solutions of APIs, the drug molecules were captured by the carriers during continuous mixing, and the complexes were separated by centrifugation from the free molecules in solution. As an alternative separation method, filtration or centrifugal filtration can also be applied. This process does not require the evaporation of the bulk solvent after complexation (freeze-drying or spray-drying usually applied for complexes of monomer cyclodextrins): only the wet product should be dried. In addition to the energy- and time-saving properties, the complexation process can be easily standardized after the determination of the loading efficiency and loading capacity of an API. To optimize the loading efficiency, the different complexation abilities of the carriers should be taken into account.

On the other hand, it is well-known that complexation of a small molecule with cyclodextrins causes a significant mass increment of the formulation due to the high molecular weight of cyclodextrins. This is especially true for cyclodextrin polymers and therefore, for oral formulations, APIs with low effective doses are required. Estradiol is suitable to fulfill this requirement as its oral dose is around 1 mg.

The biocompatibility of the cyclodextrin polymer carriers was tested by MTT test. The data show that the polymers were not toxic to Caco-2 intestinal cells after 30 min of incubation. The observed biocompatibility is in accordance with the results of Szejtli et al. whose published research show that the beads could help with wound healing and cell growth at a specified concentration [24].

The dissolution test of APIs shows that both curcumin and estradiol liberated from the complexes, but this is strongly influenced by the type of polymer. The liberated amount of APIs was low, but it should be noted that the solubility of both APIs in water is very low. We tested different dissolution buffers, but it was not possible to dissolve the APIs and obtain a concentration which is suitable for analysis. The best solution was the application of FBS, which was able to keep the APIs in solution and dissolve the molecules from the insoluble complexes. Polymer carriers did not increase the apparent permeability (P_app_) of APIs through the Caco-2 cell monolayers compared to the controls. It should be mentioned that P_app_ shows the speed of penetration, which is strongly affected by the speed of dissolution from the carriers. Indeed, P_app_ trend of APIs inversely correlated with the loading efficiency of complexes, but showed a relationship with the dissolution profiles. Lower loading efficiency caused by weaker interactions between the polymers and APIs caused better dissolution and higher P_app_ values. These observations are true for both curcumin and estradiol. In other words, carriers with lower affinity caused higher permeation, while carriers with higher affinity delayed the permeation of APIs. In both cases the permeability was lower than the control solution probably due to the retention of APIs by the polymers. As the dissolution of APIs was low from the carriers, we sought to demonstrate that estradiol was able to liberate from the insoluble complexes and reach the cellular target only by free diffusion. The results of cellular uptake studies confirmed that, though the dissolution of estradiol from the carriers was limited, complexation does not inhibit its liberation; that it reached the cellular target; and that it accumulated in the more lipophilic intracellular environment.

Taking the results together, the insoluble β-cyclodextrin polymers are suitable drug carriers for oral formulations, and the appropriate type of carrier can be fitted to the different APIs. There are two major factors which strongly influence the success of application. The first is the strength of interaction of polymers with APIs, which should be optimal for the binding and liberation of APIs. The second is the therapeutical dose of API, which should be low for the preparation of applicable oral formulations. From this point of view, estradiol is a more suitable API for a formulation with an insoluble cyclodextrin polymer carrier. The potential continuation of this work could be the design of a reservoir system that may be beneficial in the development of inhalation, subcutaneous, or possibly dermal formulations. Insoluble cyclodextrin polymers keep the drug molecularly dispersed, which protects it from environmental influences and provides a slow release from the matrix. This may be beneficial in the above-mentioned therapeutic systems.

## 5. Conclusions

Two types of insoluble β-cyclodextrin polymers were tested as possible carriers for oral drug delivery. The two polymers are chemically similar, both containing β-cyclodextrin units cross-linked by epichlorohydrin; but their appearance and physical properties are different, which affect their complexation behavior. The results showed that the size and shape of particles as well as the hydrophilic/hydrophobic character of the network have a significant role. Their advantage is the simple complexation procedure and biocompatibility, while their application is limited due to the low quantity of the complexed drugs. These carriers are only suitable for the formulation of those APIs which have low therapeutic doses.

## Figures and Tables

**Table 1:** Size of cyclodextrin polymer particles (data are presented as means ± standard deviation (SD), *n* = 20–30).

Type of Cyclodextrin Polymer	Size ± SD
βCDPB in water after 15 min	218.4 ± 38.04 µm
βCDPB in paraffin oil	154.1 ± 21.38 µm
βCDPIS in water	4.1 ± 2.5 µm

**Table 2:** The loading efficiency and loading capacity of curcumin and estradiol-glow (data are presented as means ± SD, *n* = 3).

	βCDPB	βCDPIS
Curcumin loading efficiency (%)	90.5 ± 0.2%	71.5 ± 0.5%
Estradiol-glow loading efficiency (%)	74.0 ± 4.7%	99.8 ± 0.1%
Curcumin loading capacity (‰)	0.66 ± 0.001‰	0.52 ± 0.003‰
Estradiol-glow loading capacity (‰)	1.14 ± 0.07‰	1.54 ± 0.0008‰

## Data Availability

Not applicable.

## Supplementary Materials

The following supporting information can be downloaded at: https://www.mdpi.com/article/10.3390/biom12070931/s1, Figure S1: The swelling of βCDPB in oil and water. Scheme S1: Curcumin, βCD polymers, Estradiol. Figure S2: IR spectra of pure compounds: Curcumin, Estradiol, Bead Polymer, and βCDPIS. Figure S3: IR spectra of β-cyclodextrin microbeads (Bead, synthesized in solution) and mechanochemically synthesized insoluble βCD polymer (βCDPIS). Figure S4: Simulated and measured IR spectra of physical mixtures of Curcumin-Cyclodextrin insoluble polymers. Figure S5: IR spectra of Curcumin-Cyclodextrin insoluble polymer polymer physical mixtures and complexes. Figure S6: Simulated and measured IR spectra of physical mixtures of Estradiol-Cyclodextrin insoluble polymers. Figure S7: IR spectra of Estradiol-Cyclodextrin insoluble polymer polymer physical mixtures and complexes. Table S1: IR bands (in cm^−1^) of βCD polymers, Curcumin, and Estradiol. Table S2: IR bands (in cm^−1^) of Curcumin βCD polymer physical mixtures and complexes. Table S3: IR bands (in cm^−1^) of Estradiol βCD polymer physical mixtures and complexes.
