# Supplementary material for: Investigation of the Drug Carrier Properties of Insoluble Cyclodextrin Polymer Microspheres
Source: Biomolecules. 2022 Jul 2;12(7):931. doi: 10.3390/biom12070931 (PMC9313285; doi:10.3390/biom12070931)
Supplement: Supplementary file 1 [file biomolecules-12-00931-s001.zip › biomolecules-1756112-supplementary.pdf]

Supporting Information to

Investigation of the drug carrier properties of insoluble cyclodextrin polymer microspheres

Ádám Haimhoffer[1,2,3], Alexandra Vas[1], Gabriella Árvai[1], Éva Fenyvesi[4], László Jicsinszky[5], István Budai[6], Attila Bényei[7], Géza Regdon, Jr.[8], Ágnes Rusznyák[1,2,3], Gábor Vasvári[1], Judit Váradi[1], Ildikó Bácskay[1,3], Miklós Vecsernyés[1], Ferenc Fenyvesi[1]*

[1] Department of Pharmaceutical Technology, Faculty of Pharmacy, University of Debrecen, Nagyerdei St. 98, H-4032 Debrecen, Hungary;

[2] Doctoral School of Pharmaceutical Sciences, University of Debrecen, H-4032 Debrecen, Hungary

[3] Institute of Healthcare Industry, University of Debrecen, Nagyerdei St. 98, H-4032 Debrecen, Hungary

[4] Cyclolab Cyclodextrin R & D Laboratory Ltd., Illatos St. 7, H-1097 Budapest, Hungary

[5] Department of Drug Science and Technology, University of Turin. Via P. Giuria 9, 10125 Turin, Italy

[6] Faculty of Engineering, University of Debrecen. H-4028 Debrecen, Ótemető street 2-4., Hungary

[7] Department of Physical Chemistry, University of Debrecen. H-4032 Debrecen, Egyetem Sqr. 1., Hungary

[8] Institute of Pharmaceutical Technology and Regulatory Affairs, University of Szeged, H-6720 Szeged, Eötvös u. 6., Hungary

* Correspondence: fenyvesi.ferenc@pharm.unideb.hu

Table of Contents

Figure S1	The swelling of βCDPB in oil and water.	p.2
Scheme S1	Curcumin, βCD polymers, Estradiol	p.2
Figure S2	IR spectra of pure compounds: Curcumin, Estradiol, Bead Polymer, and ßCDPIS	p.3
Figure S3	IR spectra of β-cyclodextrin microbeads (Bead, synthesized in solution) and mechanochemically synthesized insoluble βCD polymer (βCDPIS)	p.4
Figure S4	Simulated and measured IR spectra of physical mixtures of Curcumin-Cyclodextrin insoluble polymers	p.5
Figure S5	IR spectra of Curcumin-Cyclodextrin insoluble polymer polymer physical mixtures and complexes	p.6
Figure S6	Simulated and measured IR spectra of physical mixtures of Estradiol-Cyclodextrin insoluble polymers	p.7
Figure S7	IR spectra of Estradiol-Cyclodextrin insoluble polymer polymer physical mixtures and complexes	p.8
Table S1	IR bands (in cm-1) of βCD polymers, Curcumin, and 17β-Estradiol	p.9
Table S2	IR bands (in cm-1) of Curcumin βCD polymer physical mixtures and complexes	p.10
Table S3	IR bands (in cm-1) of 17 –Εστραδιολ CD polymer physical mixtures and complexes	p.11

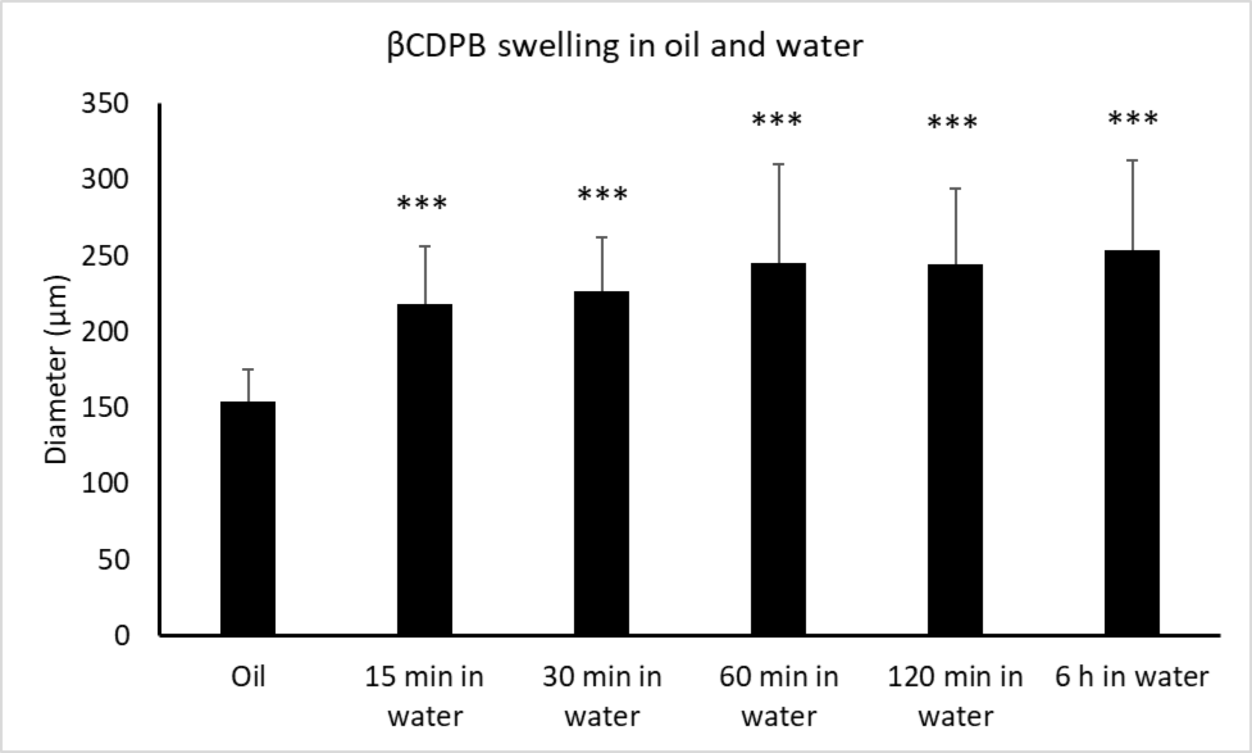

Figure S1:The swelling of βCDPB in oil and water.

βCDPB rapidly swelled in water and the mean diameter of the cyclodextrin beads significantly increased in water at different time points compared to the beads kept in paraffin oil (Data are presented as mean±SD, n=20-30; *** - p<0,001).

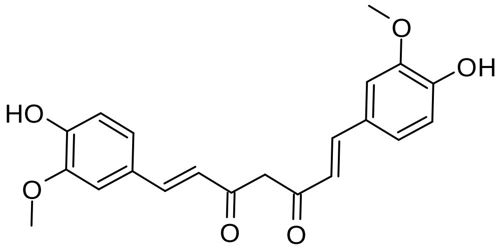

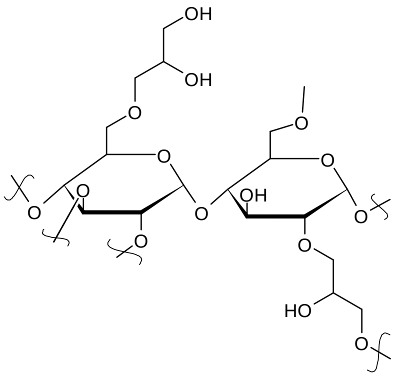

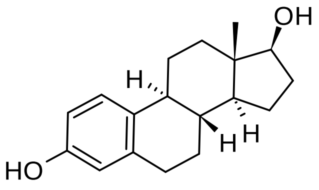

Scheme S1: Curcumin βCD polymers Estradiol

FT-IR (ATR) experiments performed on a PerkinElmer SpectrumTwo instrument, using 64 scans at 1 cm^{-1} resolution of 0.25 cm^{-1} data interval with Spectrum IR v10.2 software. Evaluation of spectra used SpectraGryph 1.2.16d software (Software for optical spectroscopy by dr. Friedrich Menges, Oberstdorf, Germany, http://spectroscopy.ninja)

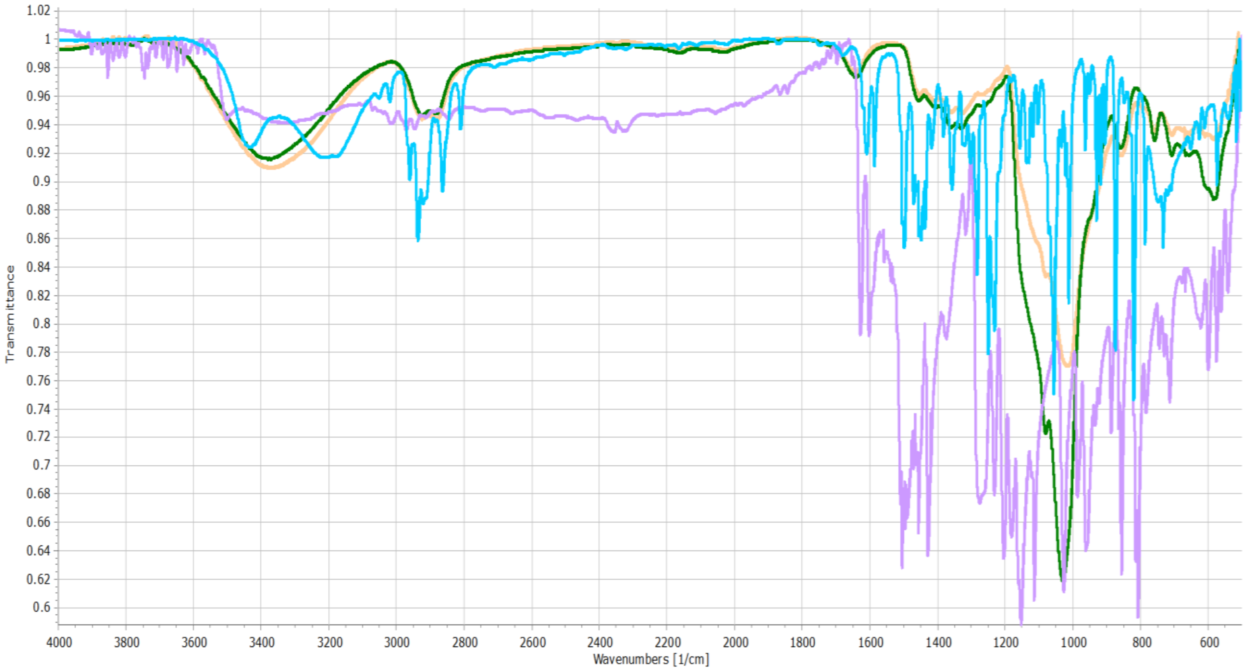

Figure S2: IR spectra of pure compounds: Curcumin, Estradiol, Bead Polymer, and ßCDPIS

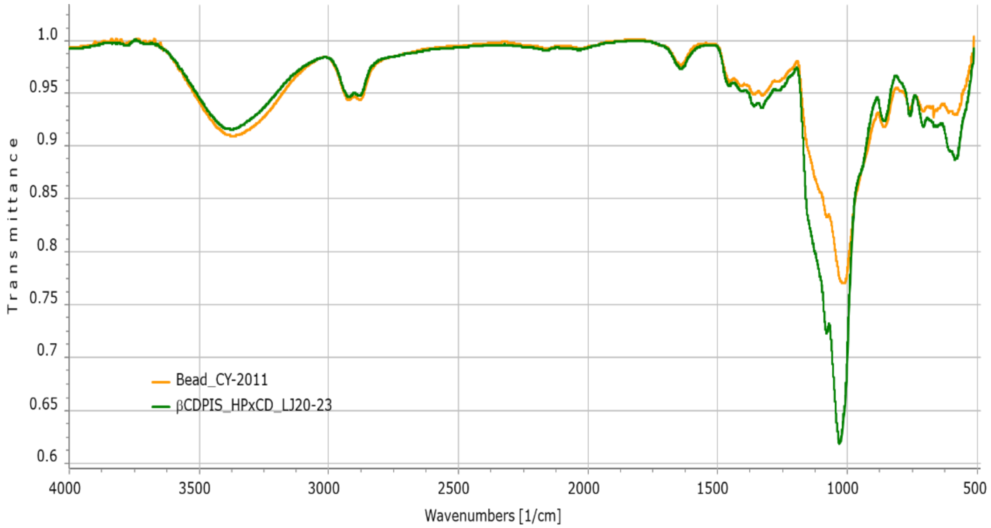

Full (500-4000 cm^{-1}) spectrum

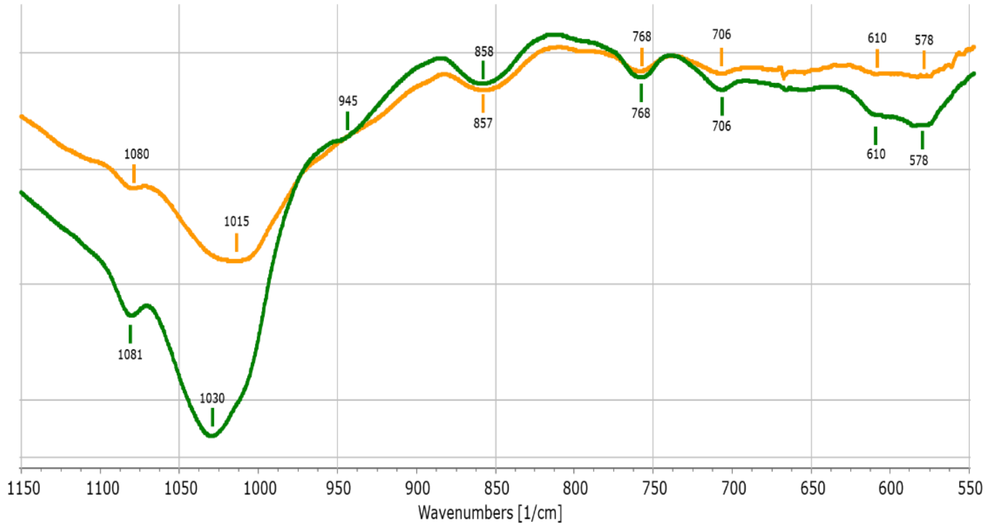

a) 550-1150 cm^{-1} region

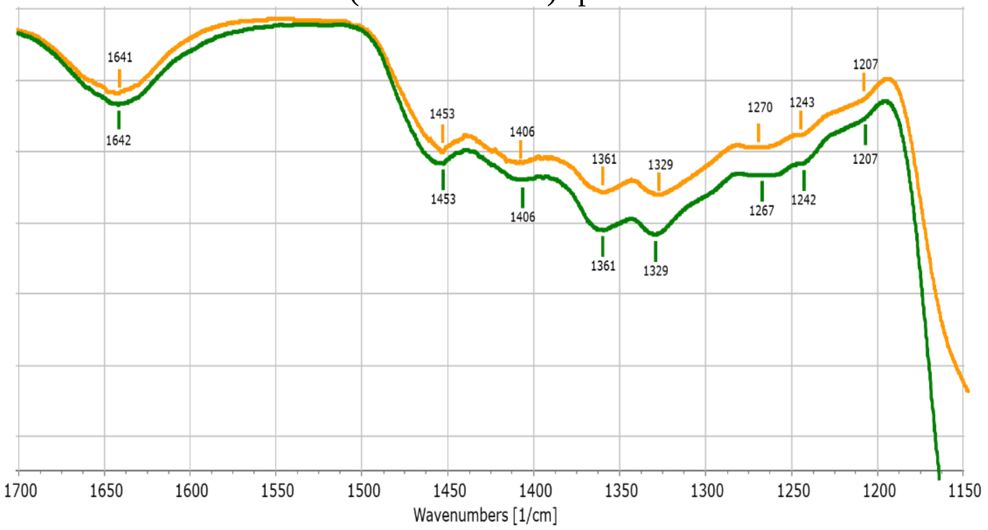

b) 1150-1700 cm^{-1} region

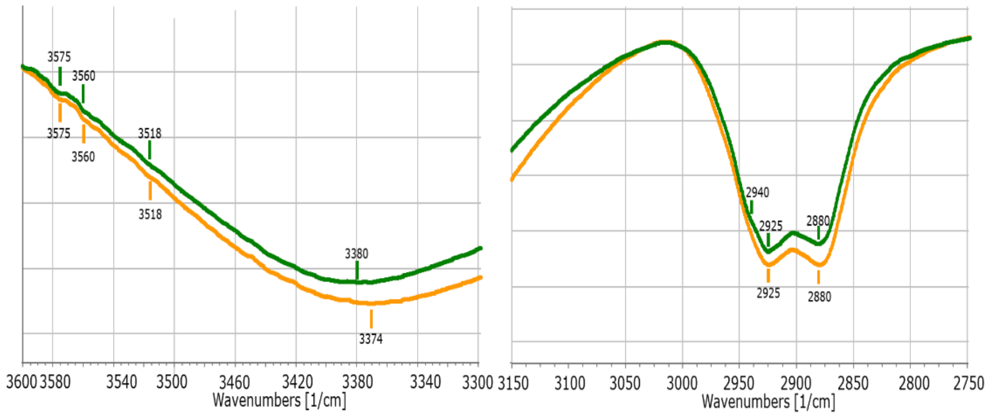

c) 2750-3600 cm^{-1} region

Figure S3:IR spectra of β-cyclodextrin microbeads (Bead, synthesized in solution) and mechanochemically synthesized insoluble βCD polymer (βCDPIS)

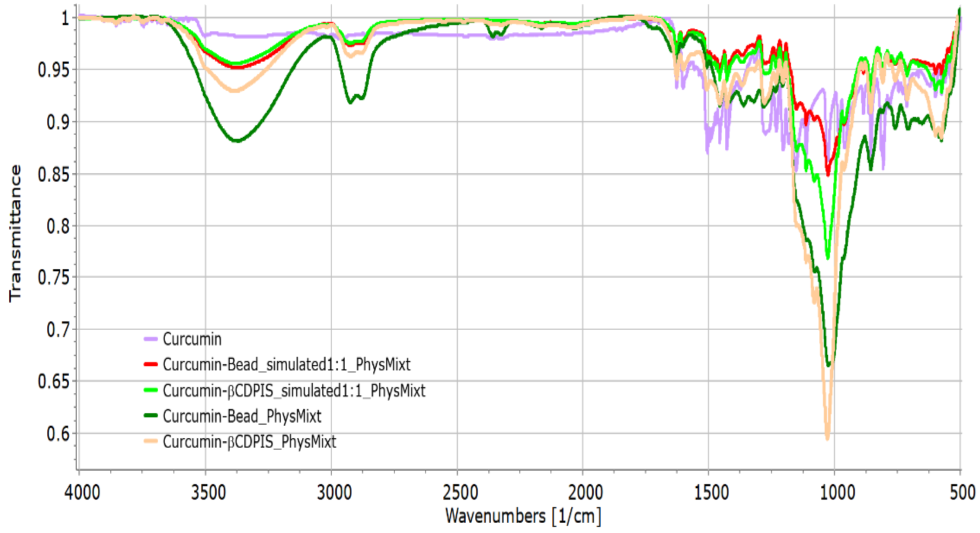

Full (500-4000 cm^{-1}) spectrum

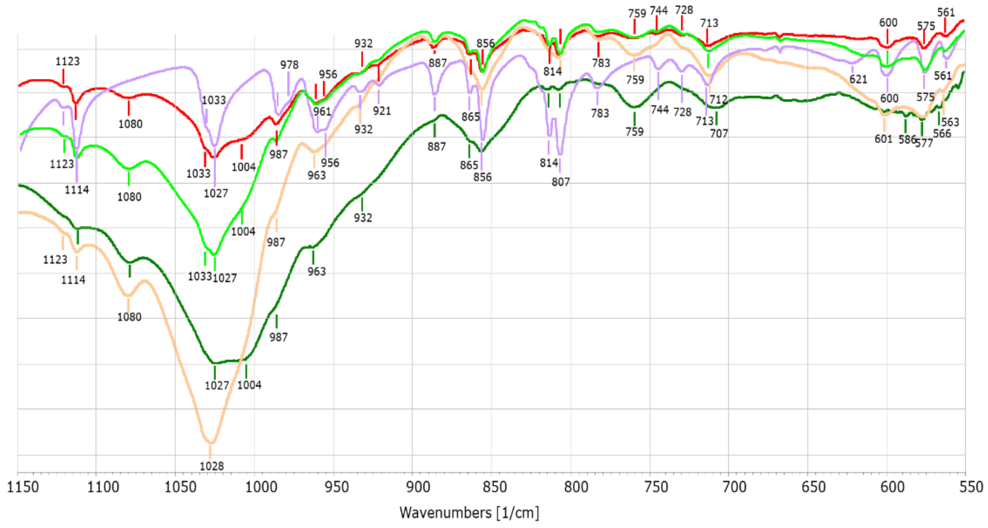

a) 550-1150 cm^{-1} region

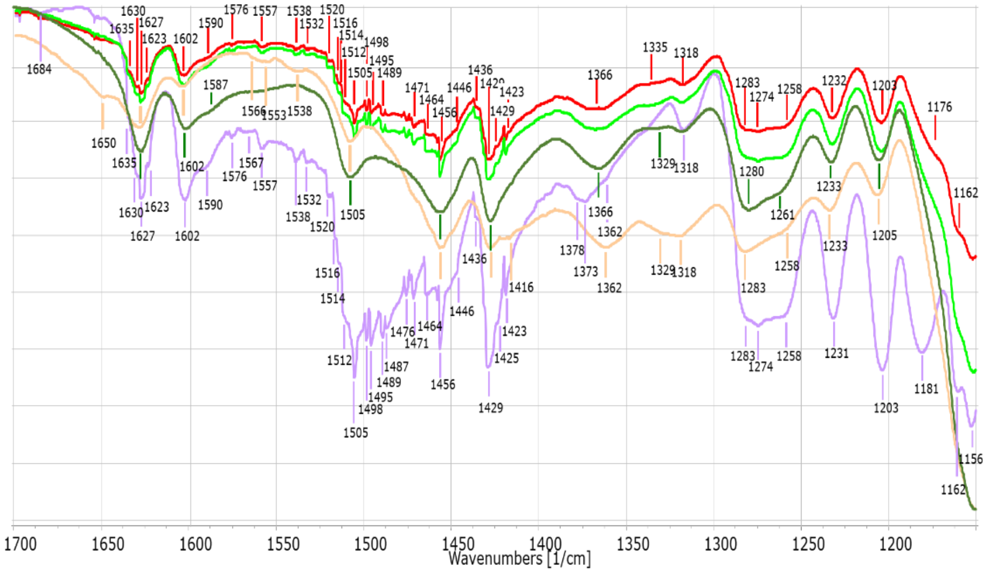

b) 1150-1700 cm^{-1} region

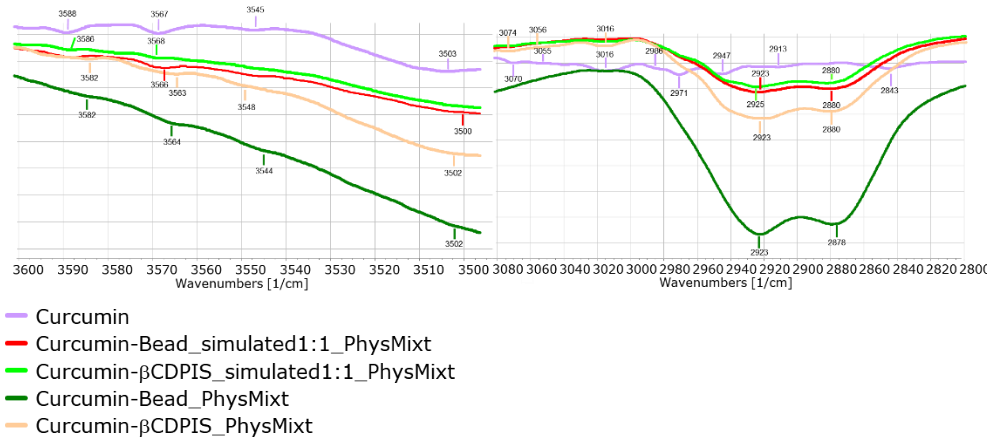

c) 2750-3600 cm^{-1} region

Figure S4: Simulated and measured IR spectra of physical mixtures of Curcumin-Cyclodextrin insoluble polymers

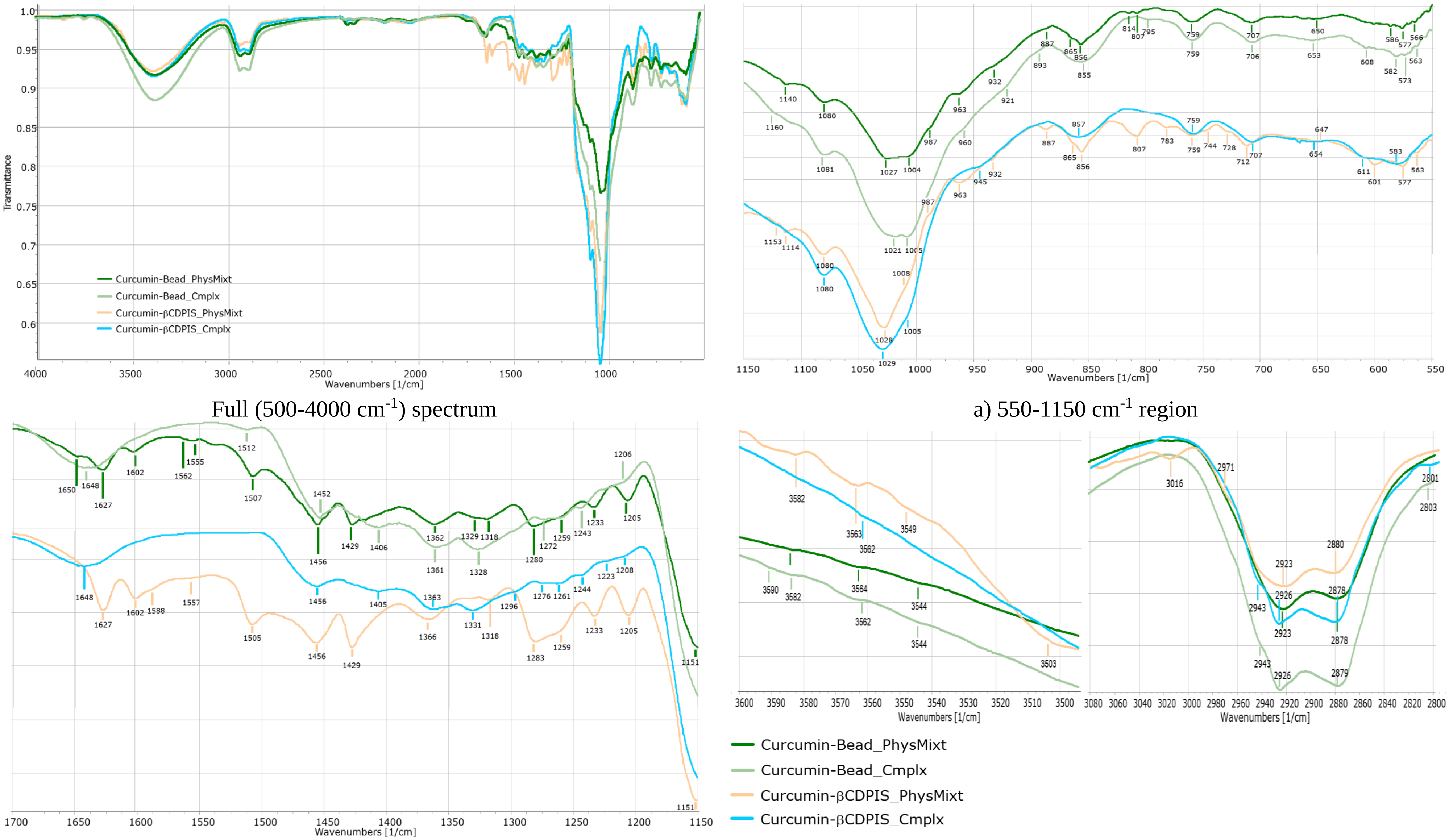

Full (500-4000 cm^{-1}) spectrum

a) 550-1150 cm^{-1} region

b) 1150-1700 cm^{-1} region

c) 2750-3600 cm^{-1} region

Figure S5: IR spectra of Curcumin-Cyclodextrin insoluble polymer polymer physical mixtures and complexes

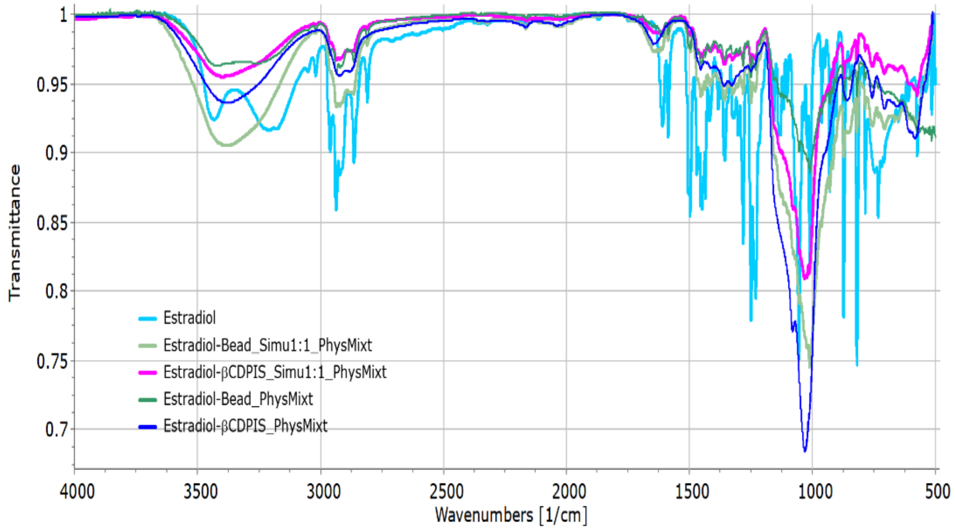

Full (500-4000 cm^{-1}) spectrum

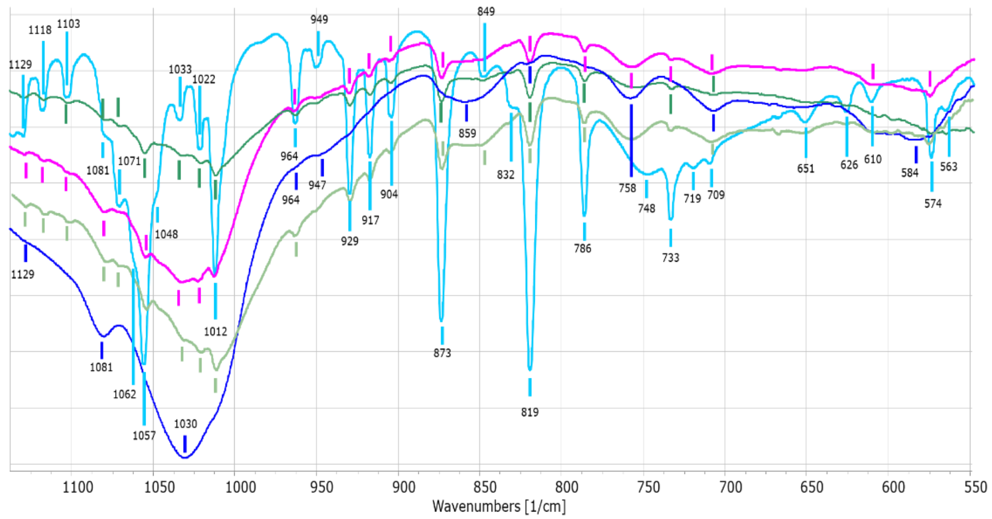

a) 550-1150 cm^{-1} region

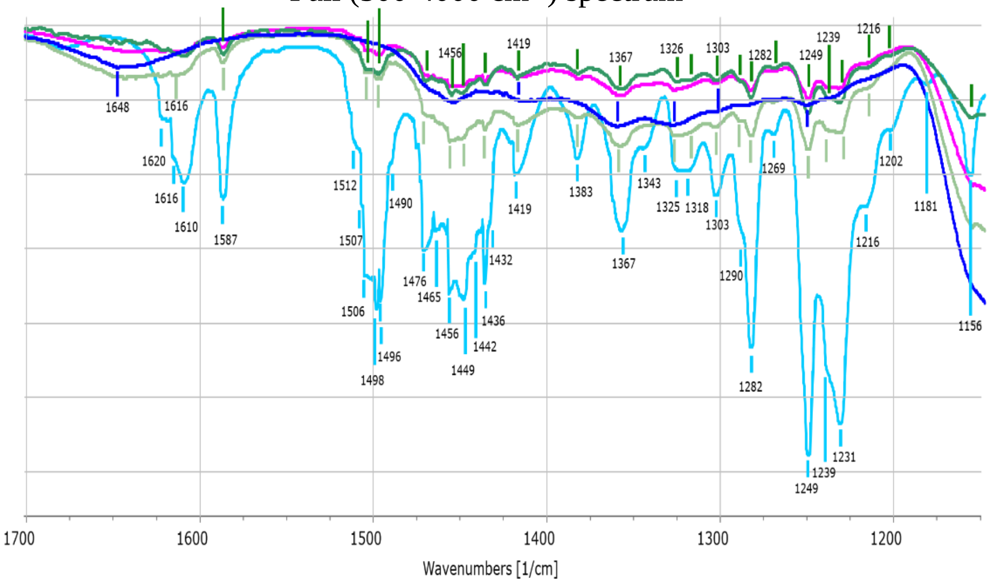

b) 1150-1700 cm^{-1} region

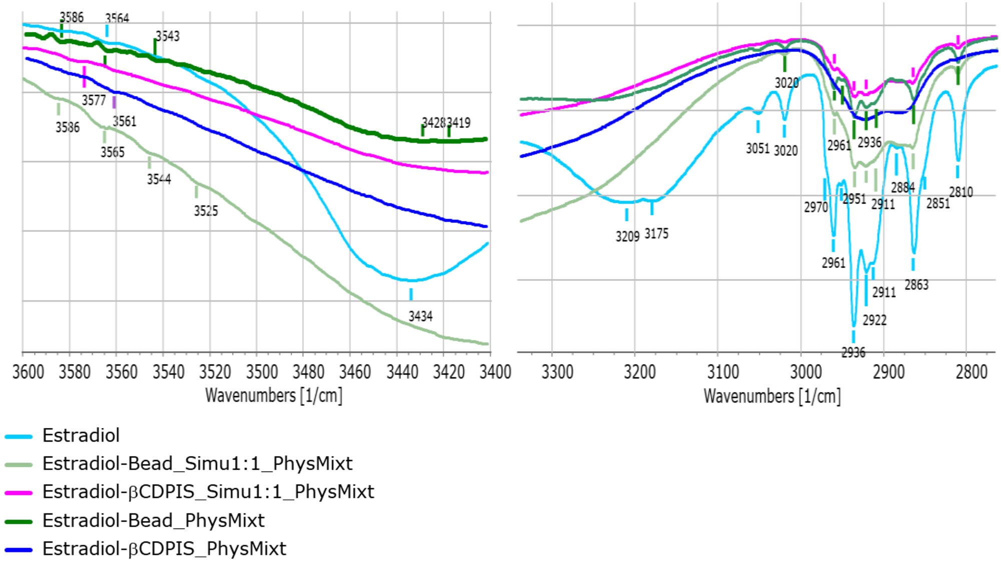

c) 2750-3600 cm^{-1} region

Figure S6: Simulated and measured IR spectra of physical mixtures of Estradiol-Cyclodextrin insoluble polymers

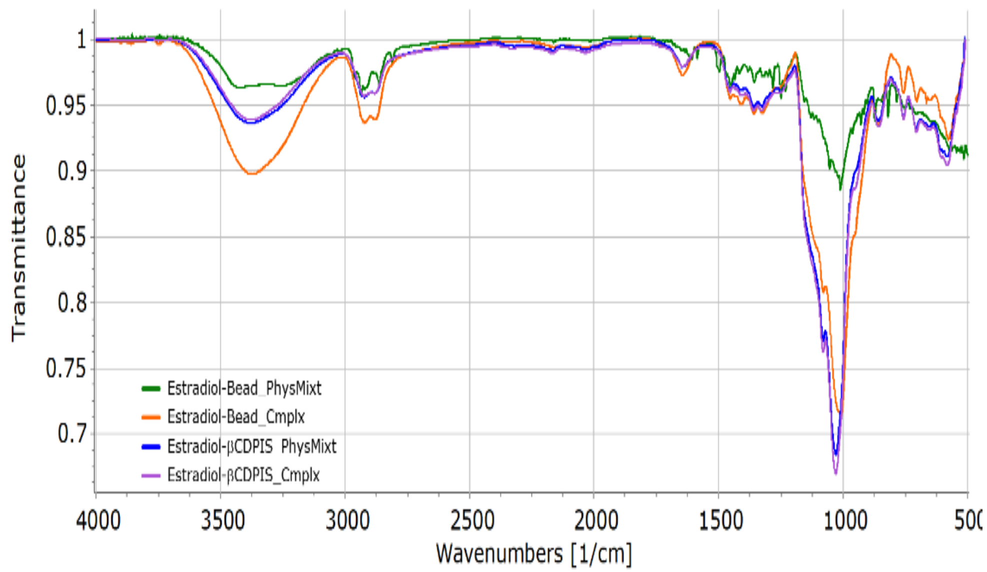

Full (500-4000 cm^{-1}) spectrum

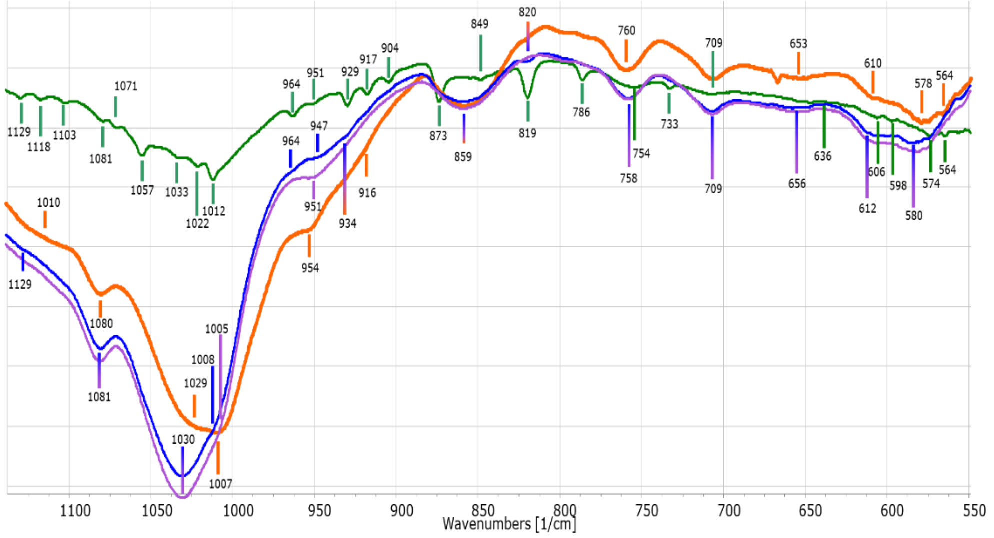

a) 550-1150 cm^{-1} region

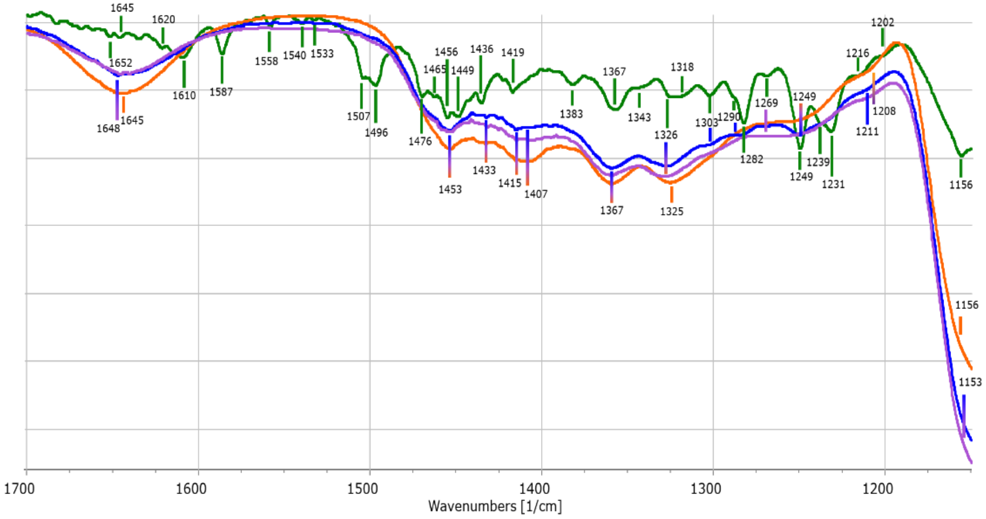

b) 1150-1700 cm^{-1} region

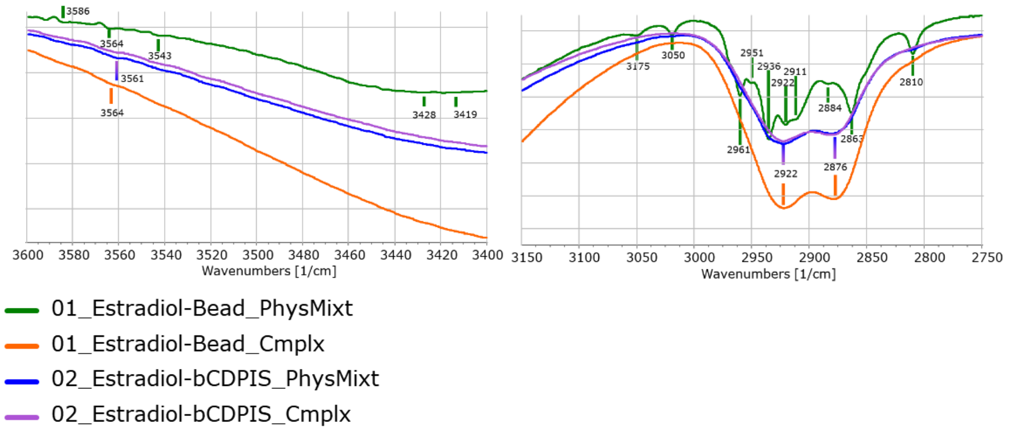

c) 2750-3600 cm^{-1} region

Figure S7: IR spectra of Estradiol-Cyclodextrin insoluble polymer polymer physical mixtures and complexes

Table S1: IR bands (in cm^{-1}) of βCD polymers, Curcumin, and 17β-Estradiol

Group	Bead	βCDPIS	Curcumin	17β-Estradiol
Phenolic O-H stretch/H-bond			3588; 3545; 3503	3586; 3543; 3434
O-H stretch/H-bond	3575; 3560; 3518; 3374	3575; 3560; 3518; 3380	3324; 3192; (3140-3480, very diffuse) *	3209; 3175; (3100-3300, very diffuse);
H-C=C-H			(3070; 3055); 3016	(3075; 3051); 3020
Aromatic C-H stretch			(3140-3480, very diffuse); (3070; 3055)	(3100-3300, very diffuse); (3075; 3051)
CH stretch	2925; 2880	2940; 2925; 2880	2988; 2971; 2947; 2913; 2843	2970; 2961; 2951; 2936; 2922; 2911; 2884; 2863; 2851; 2810
C=C-CO-R			1684	
O-C-O ring stretch	(1641)	(1642)		
Aryl-CH=CH-R			1635; 1630; 1627; 1623	
H_2O	(1641)	(1642)		
CH=CH-CH=CH			1602; (1590)	
C6-Ring/aromatic stretch				1620;1616; (1610)
Aromatic C-C ring stretch			(1590); 1576; 1567; 1557; 1538; 1532; 1520; 1516; 1512; 1505; 1498; 1495; 1489; 1487; 1476; 1471; (1464; 1456; 1446; 1429; 1426; 1423; 1416)	(1610); 1587; 1512; 1507; 1498; 1496; 1490; 1476; (1465; 1456; 1449; 1442; 1436; 1432; 1419)
CH_2 and CH_3 bending	1453; 1405	1453; 1405	(1464; 1456; 1446; 1429; 1426; 1423; 1416); 1378; 1373	(1465; 1456; 1449; 1442; 1436; 1432; 1419); 1383
C-H rock	1361; 1329	1361; 1329	1366	1367; 1343; 1325;
CH_3 bending			1318	1318
C-H/O-H in plane	1270; 1243	1267; 1242	1283; 1274; 1258	1282; 1269; 1249; 1216
Aromatic C-O stretch			1231	1239; 1231
C-O-C	1207	1207	1203	
C-O/C-C stretch	1080; 1015	1081; 1030	1156; 1033; 1027	1129; 1118; 1103; 1081; 1071; 1062; 1057; 1048; 1033; 1022; 1012
=CH-/>C-H out of plane			987; 978; (961; 956); 814; (807)	(964; 949); 904; (849; 832; 819)
C-O/C-C/C-OH bend		945	(961; 956); 932; 921	(964; 949); 929; 917
C-H	857; 768; 706	858; 768; 706	(865; 856); 728	(873; 849)
Aromatic C-H out of plane			(865; 856; 807); 783; 759; 744; 713	(849; 832; 819); 786; 748; 733;719; 709; 651
C-C-O/C-C-H	762.9; 753.1	762.9; 752.3		750.9
CH_2 rock	708.5	707.3	(712.6)	705.1
C-C-H	667.8	668	655.7	671.8

* Values in parenthesis are interchangeable or mixed of various bands

Table S2: IR bands (in cm^{-1}) of Curcumin βCD polymer physical mixtures and complexes (significant changes in red)

Group	Bead		βCDPIS	
	Phys. Mixt.	Complex	Phys. Mixt.	Complex
Phenolic O-H stretch/H-bond	3582; 3544	3590; 3582;	3582; 3549; 3503	
O-H stretch/H-bond	3564	3562	3563	3562
H-C=C-H			3016	
Aromatic C-H stretch				
CH stretch	2923; 2878	2943; 2926; 2879; 2803	2923; 2880	2943; 2926; 2878
C=C-CO-R				
O-C-O ring stretch	(1650)	(1648)		(1648)
Aryl-CH=CH-R	(1627)		(1627)	
H_2O	(1650; 1627)	(1648)	(1627)	(1648)
CH=CH-CH=CH	1602;		1602	
C6-Ring/aromatic stretch				
Aromatic C-C ring stretch	1563; 1555; 1507; (1456); 1429	1512; 1452;	1588; 1557; 1505; (1456); 1429	(1456)
CH_2 and CH_3 bending	(1456)	1406	(1456)	(1456); 1405
C-H rock	1362; 1329	1361; 1328	1366	1363; 1331; 1325;
CH_3 bending	1318		1318	
C-H/O-H in plane	1280; 1272; 1259	1272; 1243	1283; 1259	1276; 1261; 1244; (1223)
Aromatic C-O stretch	1233		1233	(1223)
C-O-C	1205	1206	1205	1208
C-O/C-C stretch	1151; 1140; 1080; 1027; 1004	1160; 1081; 1021; 1005	1153; 1114; 1080; 1028; 1008	1080; 1029; 1005
=CH-/>C-H out of plane	987; (963)	(960)	987; (963); (807)	(945)
C-O/C-C/C-OH bend	932; (963)	(960); 921	(963); 932	(945)
C-H	887; (865; 856); (707)	893; (855); (706)	887; (865; 856); (807; 728; 712)	(857; (707)
Aromatic C-H out of plane	(865; 856); 814; 807; 759	(855); 795; (759)	(865; 856); 783; 759; 744; (728; 712)	(857)
C-C-O/C-C-H	(759)	(759)	(759)	(759)
CH_2 rock	(707)	(706)	(712)	(707)
C-C-H	650	653	647	654

* Values in parenthesis are interchangeable or mixed of various bands

Table S3: IR bands (in cm^{-1}) of 17β-Estradiol βCD polymer physical mixtures and complexes (significant changes in red)

Group	Bead		βCDPIS	
	Phys. Mixt.	Complex	Phys. Mixt.	Complex
Phenolic O-H stretch/H-bond	3586; 3543; 3428; 3419			
O-H stretch/H-bond	3564	3564	3561	3561
H-C=C-H	(3175; 3050)			(3050)
Aromatic C-H stretch	(3175; 3050)			(3050)
CH stretch	2961; 2951; 2936; 2922; 2911; 2884; 2862; 2810	2922; 2876	2936; 2922; 2876	2936; 2922; 2876
C=C-CO-R	1652			
O-C-O ring stretch	(1645)	(1645)	(1648)	(1648)
Aryl-CH=CH-R	1622		(1635; 1630; 1627); 1623	
CH=CH-CH=CH			1602; (1590)	
H_2O	(1652; 1645)	(1645)	(1648; 1635; 1630; 1627)	(1648)
C6-Ring/aromatic stretch	(1610)			1620;1616; (1610)
Aromatic C-C ring stretch	(1610); 1587; 1558; 1540; 1533; 1507; 1496; 1476; (1465; 1456; 1449; 1436; 1419)	(1453; 1433; 1415)	(1453; 1433; 1415)	(1610); (1453; 1433; 1415)
CH_2 and CH_3 bending	(1465; 1456; 1449; 1436; 1419)	(1453; 1433; 1415); 1407	(1453; 1433; 1415); 1407	(1453; 1433; 1415); 1407
C-H rock	1367; 1343; 1326	1367; 1325	1367; 1326	1367; 1326
CH_3 bending/C=O stretch	1318; 1303		1318; 1303	1318
C-H/O-H in plane	1290; 1282; 1269; 1249;1239; 1231; 1216	1269; 1249	1290; 1282; 1269; 1249	1282; 1269; 1249
Aromatic C-O stretch				
C-O-C/aromatic C-O	1202	1208	1211	1208
C-O/C-C stretch	1156; 1129; 1118; 1103; 1081; 1057; 1033; 1022; 1012	1156; 1110; 1080; 1029; 1007	1153; 1129; 1081; 1030; 1008	1153;1081; 1030; 1005
=CH-/>C-H out of plane	(964; 951); (819)	(954); (820);	(964; 947); (859; 820)	(951; 859)
C-O/C-C/C-OH bend	(964; 951); 929; 917; 904	(954); 934; 916;	(947); 934	(951); 934
C-H	873; (849; 819; 786); (709)	(859; 820); (760); (709)	(859; 820); (709)	(859; 758; 709)
Aromatic C-H out of plane	(849; 819; 786); (754) ; (709)	(859; 820) ; (760) ; (709); (653)	(859; 820); (758) ; (709); (656)	(859; 758; 709); (656)
C-C-O/C-C-H	(754) ; 733	(760)	(758)	(758)
CH_2 rock	(709)	(709)	(709)	(709)
C-C-H	636	(653)	(656)	(656)

* Values in parenthesis are interchangeable or mixed of various bands
